# Effects of Enzyme Hydrolysis in Biofilm Formation and Biotic Degradation on Weathered Bioplastics

**DOI:** 10.1021/acsomega.4c10602

**Published:** 2025-04-25

**Authors:** Thomas D. Badzinski, Ariana L. Campanaro, Margaret H. Brown, Clare List, R. Lee Penn, Melissa A. Maurer-Jones

**Affiliations:** † Department of Chemistry and Biochemistry, University of Minnesota Duluth, Duluth, Minnesota 55812, United States; ‡ Department of Chemistry, University of Minnesota, Minneapolis, Minnesota 55455, United States

## Abstract

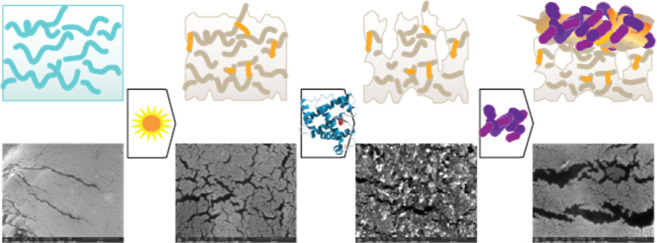

As efforts to address plastic pollution increase, new avenues are opened for the use of biologically renewable and biodegradable plastics. With the influx of these new polymer systems, it is crucial to understand the degradation processes of these polymers, particularly through disposal systems designed to manage their waste (i.e., compost). This work seeks to characterize a multistep biodegradation system by studying how enzymatic hydrolysis impacts the formation of biofilms upon weathered biodegradable aliphatic polyesters to better understand processes that should occur in composting. Poly l-lactic acid (PLLA), after varying amounts of photochemical weathering, was exposed to the esterase proteinase K followed by exposure to suspended facultative anaerobe, *Shewanella oneidensis*, whose biofilms were quantified with crystal violet staining. Enzymatic hydrolysis was observed to promote the formation of a biofilm regardless of enzymatic concentration, enzyme exposure time, and state of weathering on the polymer. This trend also held true for a less commercially viable polymer like poly(3-hydroxybutyrate-*co*-3-hydroxyvalerate) (PHBV), which was demonstrated to be resistant to enzymatic hydrolysis. Further, we observed that the state of photochemical weathering caused variable impacts to the biodegradation of PLLA. Polymer characterization suggests that while there are changes in crystallinity and surface accessible ester linkages, increased surface area caused by photodegradation and/or enzyme hydrolysis drives the observed trends. Overall, this work demonstrates a multistep biodegradation process is more effective at breaking down biodegradable polymers than a single biotic agent, though polymer weathering influences breakdown to some extent, offering insight into the importance of managing these waste streams to ensure optimal designed biodegradability.

## Introduction

The demand for the development and deployment of bioderived, biodegradable polymers is growing, making progress essential to ensure that society can continue to benefit from plastics while reducing the environmental impacts of petroleum-based materials.^1^ The successful implementation of these emerging polymer technologies relies on the ability of these “eco-friendly” materials to biodegrade as designed. Broadly, previous work states that “biodegradable” materials can be broken down into water and carbon dioxide using microbial and/or biological processes, despite biodegradable plastics being derived from biological sources (e.g., plants) or fossil fuels. Some polymers have been identified and labeled “compostable,” a subcategory of biodegradable, which means they must undergo biological processes that fully degrade into CO_2_, H_2_O, and biomass, without leaving a toxic or distinguishable residue within 180 days, as specified by engineering standards (ASTM 6400).^2,3^

Biotic degradation of polymers is a complex process, with numerous variables at play, particularly in the context of composting. The degradation process is multistep and involves the action of several enzymes such as lipases, cutinases, peroxidases, and laccases.^4^ It also requires the participation of microorganisms and biofilms.^5^ Biotic degradation can be broken down into overarching categories: biodeterioration, biofragmentation, assimilation, and mineralization. Biodeterioration is the initial step in biotic degradation where microbes (e.g., bacteria, fungi, algae, etc.) superficially degrade the polymer, leading to mechanical, physical, and chemical changes in the polymer.^6^ This step is generally accomplished by the secretion of extracellular enzymes and the attachment of microbes that lead to the formation of a biofilm.

Several studies have focused on the compostability of a variety of biodegradable plastics.^7−11^ While these studies represent successful biodegradation of biopolymers, they also represent the lack of consistency across observed studies. For example, these studies used different time frames for allowing polymers to degrade, with durations ranging from 7 days to over 100 days.^7−9,11^ Additionally, no consensus on compost properties, environmental conditions, or methods of tracking percent degradation has been established. Current standards, such as ASTM D6400 and ISO 14855, offer minimal structure for establishing and maintaining composting conditions, focusing primarily on guidelines for the end results of the composting process.^12,13^

Beyond variability in the composting conditions, few studies have investigated the biodegradation of weathered plastics, which is the inevitable condition by which these materials will enter the composting waste stream following use and environmental exposures. Weathering of plastics is the result of a variety of environmental factors, including UV-light. Photodegradation is the primary pathway of abiotic weathering and has been shown to cause changes to the molecular weight, either through cross-linking or chain scission, and crystallinity of the polymer backbone.^14−16^ These changes in polymer characteristics are synergistic, though the dominant abiotic mechanisms will vary based on the polymer backbone structure. For polylactic acid, Norrish I and Norrish II mechanisms of chain scission are the dominant photodegradation pathway and result in decreasing the molecular weight of the material including the formation of monomers (i.e., lactide) or oligomers.^17,18^

Herein, this work focuses on evaluating the biotic degradation of poly l-lactic acid (PLLA), modeling a multistep biological degradation that is more relevant to disposal conditions, such as compositing. That is, composting is a form of symbiosis, wherein various organisms interact within their substrate to degrade organic materials. For this study, enzymatic hydrolysis followed by biofilm formation is used to represent synergistic processes in the biotic degradation of the materials. PLLA is the most commonly used commercial biodegradable plastic.^19,20^ PLLA’s mechanical properties make it a suitable replacement for polystyrene, polypropylene, and polyethylene, and its industrial compostability suggests it could be a replacement for single-use plastics with a defined waste disposal pathway.^21^ Further, we use photodegradation as an accelerated aging technique to aid in our understanding of the interplay between abiotic and biotic degradation. This study provides evidence of factors that influence the biodegradation of polymers and can inform the design and use of these polymer technologies.

## Methods

### Polymer Preparation and Weathering

Poly-l-lactic acid (PLLA) and poly(3-hydroxybutyrate-*co*-3-hydroxyvalerate) (PHBV–HV content 8%; HB content 92%) thin films were purchased from Goodfellow, USA (Huntingdon, UK). Manufacturer reported specifications are shown in Supporting Information Table S1. Prior to experiments, polymer films were soaked for 24 h each in hexanes, methanol, and doubly distilled water to remove processing additives and unpolymerized monomers. Afterwhich, plastics are allowed to dry in air.

Accelerated photoaging was performed by using a Rayonet turntable photochemical reactor. Polymer films were irradiated with 16 Hg vapor lamps with photon emission centered at 300 nm (SNE Ultraviolet Co RMR-2537A). Polymer samples were irradiated for 0, 1, 2, 3, and 4 h per side. As previously reported, there is an average irradiance of 2.52 W m^–2^ for this photochemical reactor, as compared to 0.64 W m^–2^ for natural sunlight, taken at 46.7867°N on a clear June day. This correlates to roughly four times the irradiance on the polymer surface.^22^

### Enzymatic Hydrolysis Treatment

Individual samples were prepared by using a 22 mm hole punch that resulted in samples that were roughly 28 mg. Samples were soaked in 8.2 μM proteinase K (proK; Research Products International; CAS: 39450-01-6, from *Tritirachium album*) solution made in Tris HCl buffer (50 mM; pH = 7.5; CAS: 1185-53-1; Sigma-Aldrich, St. Louis, MO). These samples were then incubated for 12–36 h at 30 °C. Increasing enzymatic exposure from 12 to 36 h did not increase biofilm growth (Figure S1). Thus, we focus on results using a 12 h incubation period. Afterward, samples were rinsed with methanol and left to air-dry in ambient benchtop conditions.

### Biofilm Growth Assay

*Shewanella oneidensis* MR1 (ATCC–Manassas, VA) was inoculated on Luria–Bertani (LB) (Becton Dickinson, Sparks, MD) agar plates from frozen stock. After 24 h at 30 °C, individual colonies were transferred from the agar plates to LB broth and incubated aerobically at 30 °C with 200 rpm in a rotary incubator. Suspended bacteria were used for subsequent biofilm experiments.

Biofilms were grown on PLLA samples as previously described.^23^ Briefly, preweighed polymer films were placed in milk dilution bottles containing 19 mL of M4 nutrient-deficient broth.^24^ Samples were inoculated with 3.13 × 10^7^ cells and bottles were closed, allowing the bacteria to switch to anaerobic growth, which promotes biofilm formation.^25^ After 3 days, polymer films were removed and gently rinsed using 0.1 M phosphate-buffered saline (pH = 7.4, one part 0.2 M sodium phosphate monobasic, four parts 0.2 M sodium phosphate dibasic, five parts water) to remove planktonic cells. From here, samples were air-dried and then stained for 20 min with a 1% crystal violet solution (CAS: 548-62-9; Fisher Science Education, Nazareth, PA). The samples were removed from the stain and rinsed with deionized water, and the stain was leached from the biofilms in 3 mL of 200-proof ethanol (Decon Laboratories, King of Prussia, PA). Absorbance measurements were taken of the solutions using a Genesys 50 UV–vis Spectrophotometer (Thermo Scientific) at 595 nm to correlate the amount of biofilm grown.

### Lactic Acid Quantification with Liquid Chromatography

Polymer leachates were analyzed to determine the concentration of lactic acid released from the polymer into the bacterial M4 broth by using liquid chromatography (LC). Polymer samples were prepared by passively leaching the polymer for 3 days in bacterial M4 broth. The polymer sample was removed from the broth, and samples were prepared in LC vials while remaining broth was frozen for preservation. A Dionex UltiMate 3000 Series UHPLC (LPG-3400SD) with a 50 × 4.6 mm ID, Phenomenex Gemini C18 (3 μm particles) column was used with mobile phases including nanopure water with formic acid (0.1% v/v) and acetonitrile with formic acid (0.1% v/v). The flow rate was 0.5 mL/min for ten min, where the mobile phase was 5% acetonitrile for the first three min, then ramped to a final amount of 25%. A diode array detector was used, and chromatograms were collected and analyzed at 210 nm for lactic acid. Relative responses are reported where the signal of lactic acid was normalized to the values obtained from the pristine PLLA sample to account for a baseline shift in the samples.

### Extracellular Polymeric Substance Assay

Extracellular polymeric substances (EPS) of the biofilms were analyzed for the protein and carbohydrate concentrations. Lowry’s assay was performed on polymer samples that had undergone 3 days of biofilm growth to quantify protein content. The polymer was removed from the growth assay and gently rinsed in phosphate-buffered saline (0.1 M; pH = 7.4), Then, the sample was added to 300 μL of nanopure water in a 15 mL falcon tube. Subsequently, 1.5 mL of alkaline copper reagent (1 mL 2% (w/w) NaKC_4_H_4_O_6_ (CAS: 6381-59-5; Fisher Chemical, Fair Lawn, NJ), 1 mL 1% (w/w) CuSO_4_·5H_2_O (CAS: 7758-99-8; Acros Organics, NJ), 98 mL of 2% (w/w) NaHCO_3_ (CAS: 144-58-8, Fisher Chemical, Fair Lawn, NJ) in 10 mM NaOH, and 75 μL of Folin–Ciocalteu reagent (Spectrum, Gardena, CA) was added. The mixture was then gently vortexed for 5 min and incubated at room temperature for 30 min. Absorbance of the solutions was measured at 500 nm. Bovine serum albumin (BSA) standards were used with the assay to create a calibration curve for quantification.

A phenol-sulfuric acid assay was performed on the biofilm samples to quantify carbohydrates in the EPS. Polymer samples, different from those used in protein quantification, were removed from the growth assay and gently rinsed in phosphate-buffered saline (0.1 M; pH = 7.4). Then, the sample was added to 500 μL of nanopure water in a 15 mL falcon tube. The polymer itself was shown to react with the reagents. Therefore, the samples were sonicated in a Branson 2510MT Ultrasonic Cleaner for 10 s pulses to a total of 60 s, which releases the EPS while leaving the cells largely intact and the polymer was removed from the assay.^26^ 50 μL of phenol (91%) (CAS: 108-95-2; Fisher Chemical, Fair Lawn, NJ) was added, along with 5 mL of concentrated H_2_SO_4_ (95.5%) (CAS: 7664-93-9; Columbus Chemical, Columbus, WI). This mixture was then gently vortexed, incubated at 35 °C for 20 min, and left to stabilize at room temperature for 4 h. Absorbance of the solutions was measured at 480 nm. Glucose standards were used with the assay to create a calibration curve for quantification.

### Polymer Characterization

#### Attenuated Total Reflectance-Fourier Transform Infrared Spectroscopy

ATR-FTIR (Nicolet iS50 ATR-FTIR; Thermo Fisher) was performed to characterize molecular changes that occurred on the polymer surface. Spectra were collected at a minimum of 3 locations per sample with an average of 64 scans at a 4 cm^–1^ resolution. Igor Pro 8.04 was utilized to integrate bands in regions of interest within the individual spectra. The regions of interest were the ester (1150–1250 cm^–1^), carbonyl (1710–1810 cm^–1^), vinyl (1600–1700 cm^–1^), and hydroxyl (3000–3600 cm^–1^) regions and a reference band of ∼1455 cm^–1^ was used. Regions of interest were normalized to the reference region and converted to a ratio (). Samples were measured in triplicate, with the mean and standard deviation reported.

#### Differential Scanning Calorimetry

DSC measurements were conducted using a TA Instruments DSC 250+ calorimeter as previously described Brown et al. with a heat–cool–heat cycle from 25–170 °C and a ramp rate of 10 °C/min.^16^ Samples were prepared in triplicate, using between 2 and 10 mg of the irradiated polymer in a Tzero pan. Crystallinity analysis was performed for the first heat cycle. Enthalpy of melting was used to determine sample crystallinity where enthalpy was normalized to the enthalpy of melting of a 100% crystalline sample (PHBV—109 J/g, PLA—93.6 J/g) to yield percentage bulk crystallinity.^27,28^

#### ^1^H NMR Analysis

End member analysis using ^1^H NMR was performed as was described in Pérez et al., where integrating the quadruplets for the end group methine (i.e., bonded to the acid) versus the internal methine group (i.e., bonded to the ester) provides a ratio that represents repeating units.^29^^1^H NMR was performed on Bruker Advance 400 MHz NMR using CDCl_3_ as a solvent.

#### Scanning Electron Microscopy

Scanning electron microscopy (SEM) images were obtained by using a Thermo Apreo 2S Lo-Vac scanning electron microscope. Plastic films were secured onto aluminum stubs (Structure Probe, Inc.) using conductive carbon tape and sputter-coated with 5 nm of platinum (Leica EM, ACE 600) before analysis. A 5.0 kV accelerating voltage and a working distance of 10 mm were used during the imaging.

## Results and Discussion

### Relating Abiotic and Biotic Degradation Treatments to Biofilm Growth

**Figure 1:**
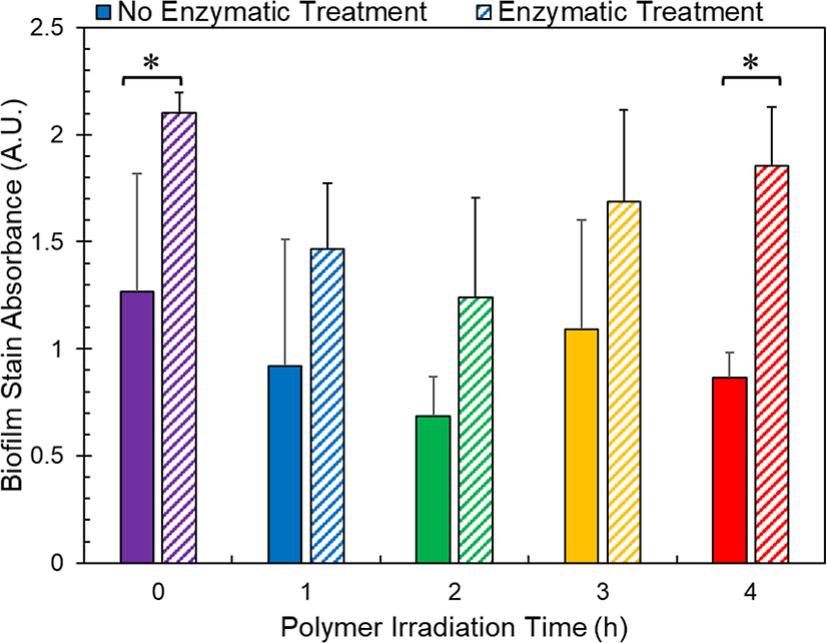
Bar graph showing biofilm growth on irradiated PLLA samples as quantified using crystal violet staining. The bars represent the average (*n* = 3), and the error bars represent the standard deviation. ANOVA analysis performed on no enzyme (*p* = 0.508) and enzyme treated (*p* = 0.036) samples as it relates to irradiation time. *t*-test performed comparing means of same UV-light doses between no enzyme and enzyme treated group with *p < 0.05.

Seeking to model biotic degradation with a more relevant multistep process, we observed that the changes imparted to the polymer surface by the enzymes result in a more amenable environment for the formation of biofilms. Figure 1 shows the amount of biofilm on PLLA samples that have been treated with UV light and proK incubation. First, we see promoted biofilm formation, as shown by higher stain uptake and release from the samples, across all the stages of photodegradation as the result of the enzymatic pretreatment, though only 0 and 4 h samples demonstrate a significant difference between nonenzyme and enzyme treated samples. Previous work suggests that this could be the result of irreversible binding of protein to the surface making a more amenable surface for bacterial binding.^30−32^ However, experiments with nonenzymatic protein pretreatment (i.e., with bovine serum albumin (BSA)) did not support this observation as BSA treatment did not increase the amount of biofilm (Supporting Information Figure S2). Autohydrolysis of the surface that results from PLLA being in solution may also influence surface structure of the plastic and subsequent attachment of bacteria; however, the autohydrolysis observed in a previous study^16^ and shown in Supporting Information Figure S24 would equate to <0.5% of the transformation compared to the enzyme-effect. We concluded that the changes imparted to the polymer by the enzymes, rather than the enzymes themself, result in a more amenable surface for the bacteria.

Weathering of polymer changes polymer characteristics, such as crystallinity, surface area, molecular weight, and accessible ester linkages, ultimately influencing the compostability of a polymer. Using UV light to accelerate weathering, we observed that biofilm growth did not have a trend with increasing irradiation time, where the 0 h PLA sample had higher biofilm growth than the 1 and 2 h samples. However, the growth recovers to control levels for the polymers weathered with ultraviolet (UV) light for 3 and 4 h. Previous work has demonstrated that greater photodegradation yields greater amounts of plastic cleaved by enzymatic hydrolysis.^16^ Additionally, it has been recently established in the literature that UV irradiation promotes biofilm formation within nonbiodegradable systems.^33,34^ We hypothesize that a combination of polymer changes the 1 and 2 h UV light treated samples, such as crystallinity, surface area, molecular weight, and accessible ester linkages (i.e., sites of enzymatic hydrolysis), preventing bacteria from attaching and/or propagating on the surface.

### Characterization of Biofilms’ Properties

To better understand the characteristics of the biofilms that formed on the UV and enzymatically treated PLLA, the EPS of the biofilm was analyzed. The amount of protein in the biofilm EPS is significantly higher in samples that received an enzymatic pretreatment (Supporting Information Figure S3). Similarly, carbohydrate concentrations are higher in the enzyme treated samples, although not statistically significant. These results are likely due to the increased biofilm present on the enzymatically treated samples. It has been previously reported that larger biofilm growths will have a larger amount of EPS.^35^

**Figure 2:**
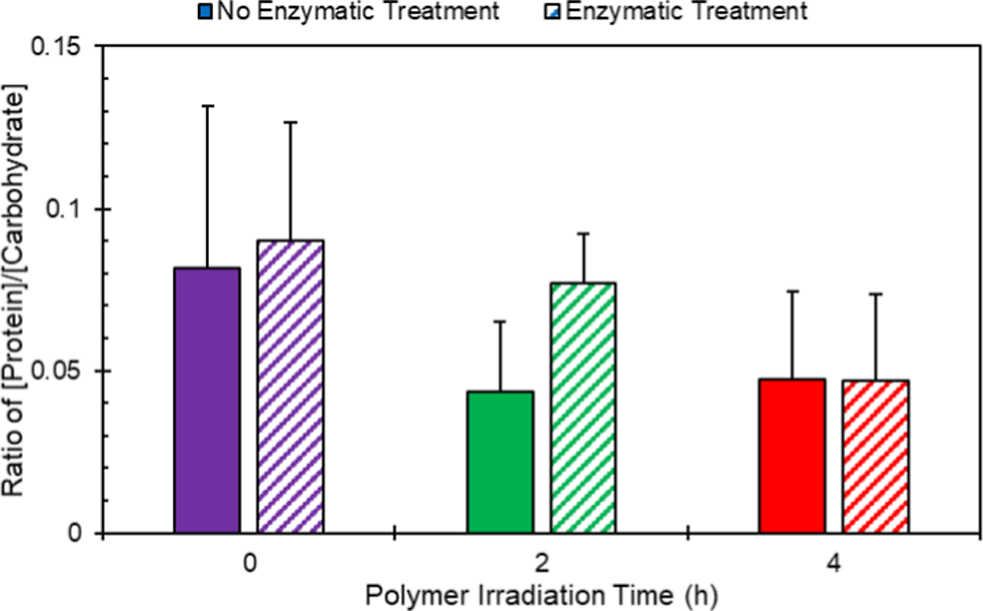
Ratio of the protein to carbohydrate concentration of nonenzymatic and enzymatic treated PLLA samples at varying degrees of photodegradation. Triplicate concentrations were averaged, and error was propagated through the standard deviations of the protein and carbohydrate concentrations.

Considering the ratio of protein to carbohydrates, we observed a steady decrease with increased photoweathering (Figure 2). While increased abundance in proteins and carbohydrates is expected with increased amounts of biofilm, the ratio of protein to carbohydrate concentrations can speak toward variability in the biofilm characteristics. This trend is driven by the steep increase in carbohydrates present, whereas the protein concentration stays relatively constant (Supporting Information Figure S3B). This increase in the carbohydrate concentration with increasing UV aging time is likely due to a combination of two factors. First, it has been established that carbohydrates affect the “stickiness” or cohesion factor of the matrix.^35^ As a result of the superficial degradation of the polymer through UV aging, the bacteria are producing more carbohydrates to increase its ability to stick to the degraded surface. Second, it has been established that lactic acid, as well as monomer units of other polymers, can be utilized by bacteria as a sole carbon source.^36−38^ This increase in carbohydrate may also be attributed to the metabolism of chain scission products.^39^ Interestingly, there are minimal differences in the ratio due to enzyme pretreatment for biofilms with identical irradiation times (i.e., solid compared to striped bars). This suggests that photodegradation is the largest driver in the bacterial response to the surface properties.

### Planktonic Bacteria Response to Solution Conditions

**Figure 3:**
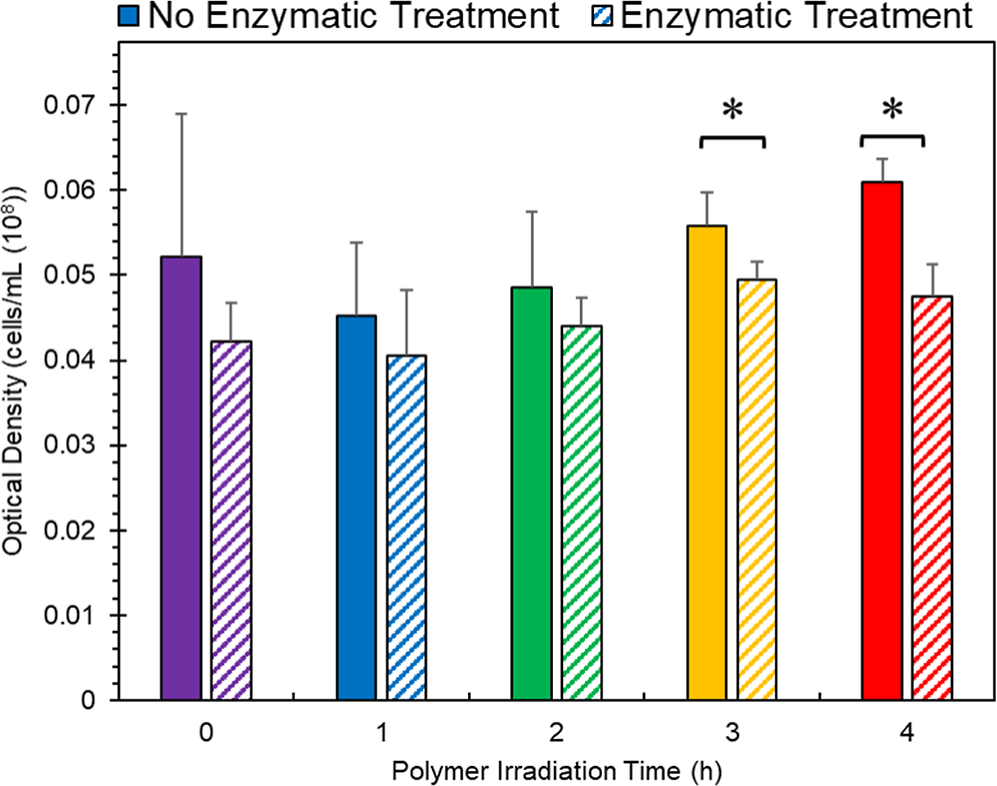
Optical density at 600 nm of remaining M4 nutrient broth after 3 days of biofilm growth. Bars represent the average (*n* = 3) of the samples with error bars representing standard deviation. ANOVA analysis performed on no enzyme (*p* = 0.213) and enzyme treated (*p* = 0.088) samples as it relates to irradiation time. *t-*test performed comparing means of same UV-light doses between no enzyme and enzyme treated group with *p < 0.05.

Investigating the number of planktonic cells remaining in the broth after the polymer samples were removed allowed for exploration of the nutrient competition between planktonic cells and biofilm growth (Figure 3). Unlike the biofilms, the nonenzymatically treated bacteria had uniformly increased amounts of suspended bacterial cells compared to the enzymatically treated samples, as measured by solution optical density (OD). Additionally, it was observed that the number of suspended cells increased as irradiation time increased. This potentially means that digestible monomer, dimer, and oligomer units were leached from the polymer for the planktonic bacteria or that the bacteria themselves are releasing digestible degradation products into the broth. We hypothesized that one reason the enzymatic treated samples had lower planktonic cell counts was the result of soaking samples in the enzyme-buffer solution before moving to the biofilm growth system, therefore removing the leachable food for the bacteria. To assess this possibility, an additional assay was performed where polymer samples were soaked in Tris HCl buffer (50 mM; pH = 7.5) for 12 h without enzyme present then moved to the biofilm growth chambers. The biofilm OD measurements of buffer-soaked samples closely resemble the nonenzymatic data (Supporting Information Figure S4). This suggests that the impact of the enzyme pretreatment is not limited to easy-to-leach small molecules providing food for the planktonic bacteria. However, presoaking in buffer shows a variable effect to the biofilm formation as it relates to UV degradation times (Supporting Information Figure S5). One likely explanation for enzymatic pretreatment leading to a lower planktonic growth could be that as the polymer surface is changed through UV aging and enzymatic hydrolysis, bacteria move to form a more abundant biofilm rather than continue planktonic growth. This may explain the decrease in planktonic cell growth while the biofilm abundance is higher overall.

### Characterizing Solution for Monomer Release

**Figure 4:**
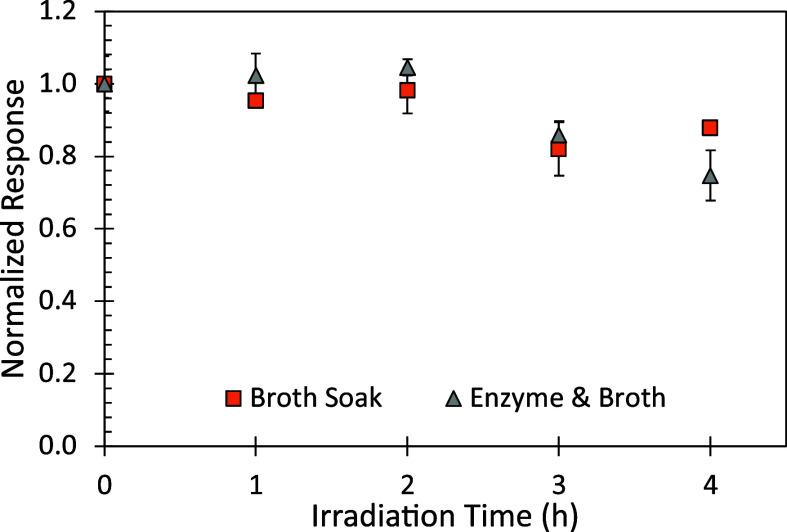
Leached lactic acid from PLLA samples in M4 broth as quantified with LC versus UV light degradation time and normalized to the values of the 0 h (pristine) sample. Markers represent the average (*n* = 3) with the error bars representing the standard deviation. “Broth Soak” are plastic samples that are similar to those in the nonenzymatically treated polymer sample whereas “Enzyme and Broth” are the samples in the enzymatically pretreated sets.

We observed that the concentration of lactic acid monomers in broth relates to the UV degradation state of the polymer, with decreasing amounts of lactic acid in the solution as irradiation time increases (Figure 4). These trends were the same for the solutions that mimic the nonenzymatic and enzymatic growth conditions. This contradicts the previously mentioned speculation that the heavily UV aged polymer samples were leaching an increased amount of degradation products, one of which has been demonstrated to be the monomer lactide, that may be providing sustenance for the planktonic cells.^17,18,40^ One explanation for the trends observed in lactic acid leaching is that monomer formation would be the result of chain scission occurring at polymer end groups, though random chain scission is far more likely. Therefore, the leached small molecules are more likely oligomers rather than monomers, which would occur more readily with the UV-light-induced scission at the maximum irradiation. Therefore, planktonic growth being promoted may be related to oligomer release or, alternatively, the release of molecules in the exudate (i.e., EPS) from the bacteria themselves, rather than the PLLA.

In summary, the enzymatic pretreatment of PLLA promotes the attachment of bacterial biofilms. This is the result of the enzymes creating a more amenable surface for biofilm formation, potentially through the increase in bindable surface area. It was also observed that UV irradiation-induced changes in the polymer surface play a key role in the ability of bacteria to form a biofilm. Further, EPS characterization suggests that the bacteria produce different extracellular components to respond to the changing surface. Additional changes to EPS composition may result from the metabolization and release of bacterial degradation products into solution. This is evidenced by the delayed, sustained growth observed in a biofilm kinetics assay (Supporting Information Figure S6) and the overall decrease in protein to carbohydrate ratio, where carbohydrate content increased. This increase in carbohydrate is not related to the increase in UV aging providing more degradation products, as LC indicates that there is less leached lactic acid as UV aging increases. Overall biotic degradation is furthered by the presence of extracellular enzymes coupled to bacterial communities, which is the foundation of the symbiotic relationships observed in composting systems. However, it should be noted that the state of the polymer plays a key role in the ability of the system to biologically degrade.

### Polymer Characterization–Surface Chemistry

**Figure 5:**
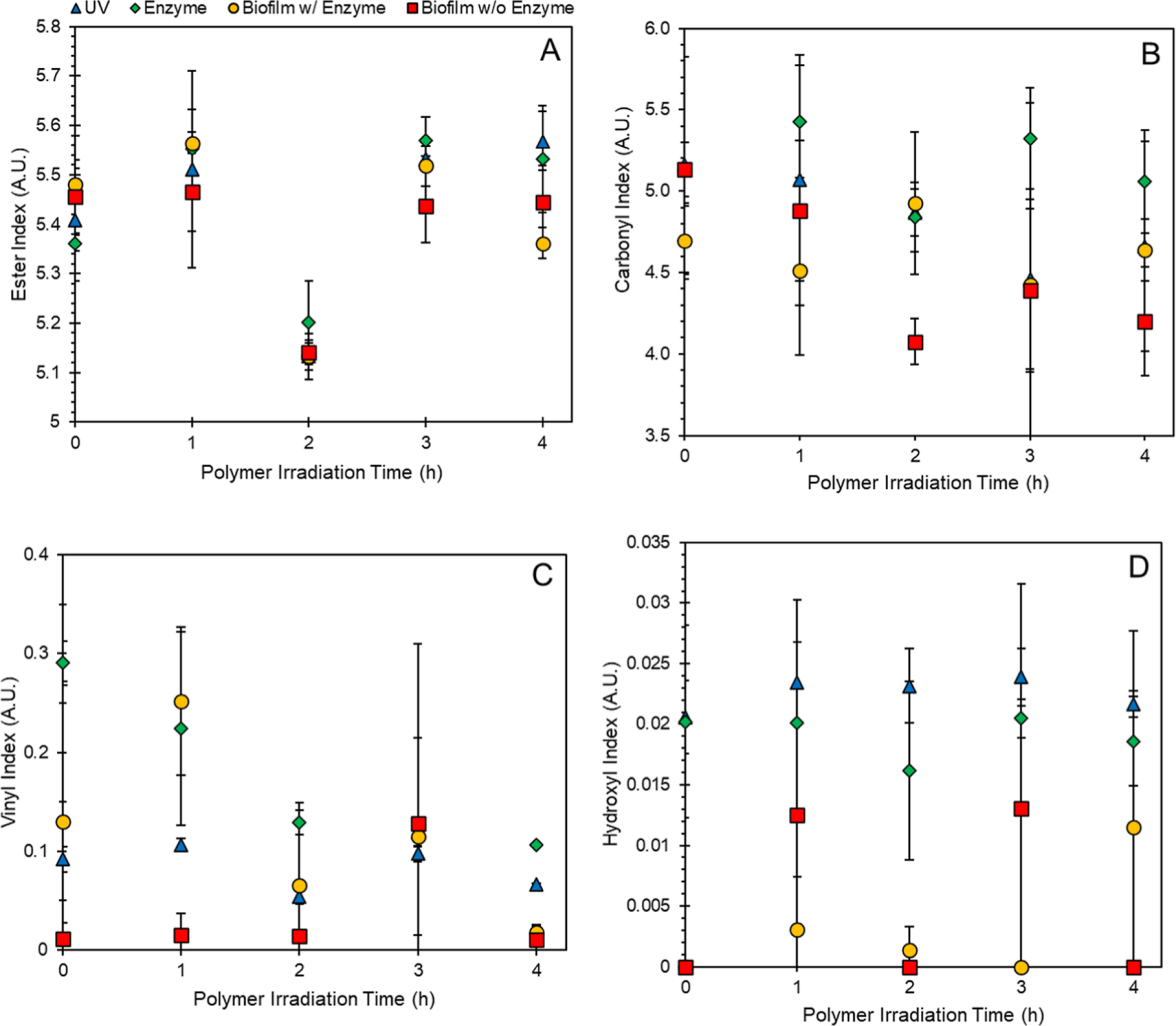
(A) Ester (1150–1250 cm^–1^), (B) carbonyl (1710–1810 cm^–1^), (C) vinyl (1600–1700 cm^–1^), and (D) hydroxyl (3000–3600 cm^–1^) indices of irradiated PLLA samples at varying points in the degradation process. Samples were measured in triplicate and averaged with error bars representing the standard deviation. UV: irradiation, enzyme: irradiation and 12 h in 8.2 μM proK, biofilm w/enzyme: irradiation, 12 h in 8.2 μM ProK and 3 days inoculated with *S. oneidensis*, biofilm w/o enzyme: irradiation and 3 days inoculated with *S. oneidensis*.

Applying the various abiotic and biotic degradations to PLLA results in very minimal changes to the surface chemistry, as demonstrated by FTIR characterization. Figure 5 (Supporting Information Figures S12–S15) shows ester (1150–1250 cm^–1^), carbonyl (1710–1810 cm^–1^), vinyl (1600–1700 cm^–1^), and hydroxyl (3000–3600 cm^–1^) indices, where the absorbance of a band from a particular bond is normalized to the reference band at 1455 cm^–1^ (FTIR spectra shown in Supporting Information Figures S7–S11). This has previously been reported by Campanaro et al., who demonstrated that photodegradation and incubation in sludge induce minimal chemistry changes to photodegraded PLLA.^41^

One exception in our data was the hydroxyl index, where the plastic samples in which biofilms were grown had lower OH stretches. This could be due to the metabolism of terminal OH groups by the biofilm matrix as hydrophilic polymers like PLLA have a microbial affinity allowing for favorable microbial adherence and a rapid change in surface roughness in comparison to hydrophobic polymers.^42^ It should be noted that this trend could also be the result of residual bacteria on the films obscuring the signal. However, since this trend was not observed on other indices, we do not believe this to be the main driving factor.

While minimal changes were observed in the chemistry overall from either abiotic or biotic degradation, an interesting irregularity was observed in the ester index within the 2 h polymer sample across all biotic treatments. This irregularity is similar to the one observed in the biofilm assay and could point to a reason for the observed trends in biofilm growth, where the 2 h UV light irradiated sample had lower bacterial growth (Figure 1). This supports the premise that superficial ester linkages are necessary for biofilm attachment. While this has not been explicitly reported in the literature, there are several studies discussing similar peak photoirradiation time frames where biotic degradation is highest. In one study, 8 h PLLA shows the highest biotic degradation, where further time points like 12, 16, and 24 h show reduced biotic degradation. It was theorized that the reduction in biotic degradation caused at further time points was due to the PLLA becoming more recalcitrant due to excess aging turning the polymer into a brittle, white solid that could not be assimilated by bacteria.^43^ This may also be the result of conditions where surface wettability, accessible chain lengths, and/or degradation products are nonoptimal for bacterial growth and assimilation.^44,45^ This combination of surface wettability, accessible chain length, and nonmetabolizable degradation products together likely create an environment at the 2 h time point that is not conducive for bacterial growth.

### Bulk Crystallinity and Molecular Weight

**Figure 6:**
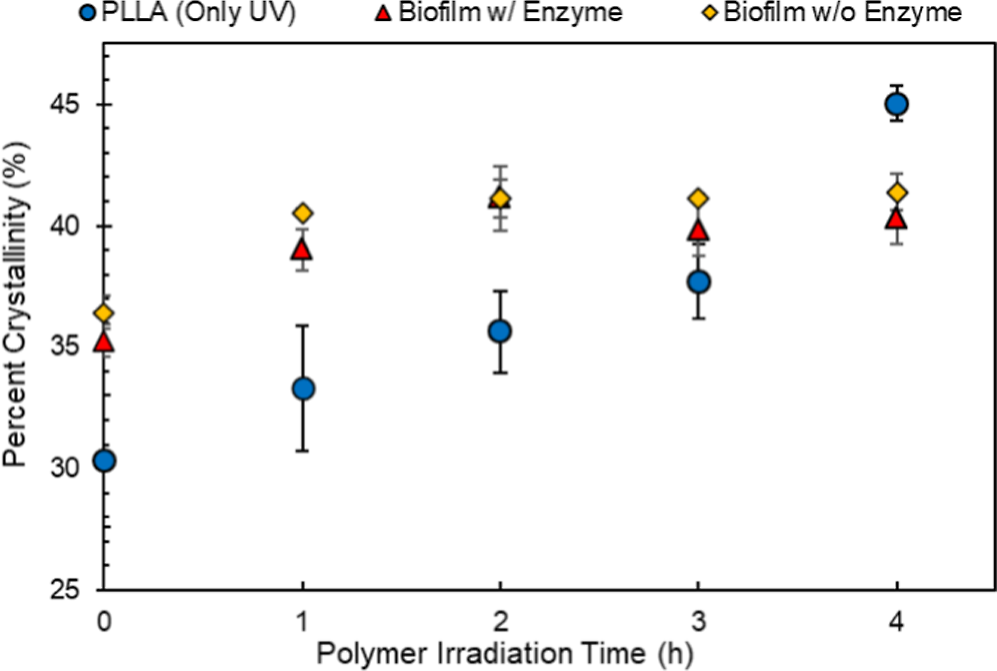
Percent crystallinity (%) versus UV light irradiation time of PLLA with differing treatments using DSC. Markers represent the average of samples (*n* = 3) with error bars representing standard deviation.

The bulk crystallinity of photodegraded PLLA increased with irradiation time for all treatments (Figures 6 and Supporting Information Figure S17). Within the samples that received varying degrees of biological treatment (enzyme or biofilm growth), there is an apparent increase in crystallinity in comparison to that of samples that only received UV treatment. We hypothesize this trend is likely due to the hydrolysis of the amorphous region, by either the bacteria and/or enzyme, within the polymer, resulting in a higher fraction of crystalline polymer remaining post-treatment. While this trend could also be an artifact of the time the polymer spends in aqueous conditions during the biological degradation disrupting the thermal history, evidence in the literature does not support water causing solvent-induced crystallization.^46^ End member analysis of the photodegraded samples revealed a quantitative decrease in the molecular weight of PLLA as a result of the photodegradation (Supporting Information Table S2 and Figure S16). Biodegradation also causes the molecular weight of the samples to decrease (Supporting Information Table S2). Further analysis of the temperature where there is a maximum enthalpy change during melting (i.e., the temperature of the endothermic peak), which is related to the molecular weight of the polymer, reveals the molecular weight of the remaining plastic is higher after biological treatment (Supporting Information Figure S18), further supporting that enzyme and biofilm growth preferentially degrade the shorter and amorphous polymer chains. This is a well-established phenomenon in the literature and may also be the driving force in the decrease in biofilm growth within the intermediately irradiated samples, as crystallinity is one of the factors inversely related to polymer biodegradation.^47,48^

### Polymer Surface Morphology

**Figure 7:**
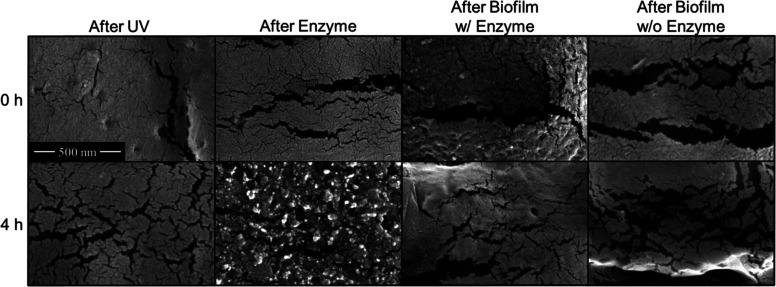
SEM images of PLLA with no or 4 h UV light irradiation and exposed to varying biological degradation treatments. Conditions are as follows, UV: irradiation with 300 nm light, enzyme: irradiation and 12 h in 8.2 μM proK, biofilm w/enzyme: irradiation, 12 h in 8.2 μM ProK and 3 days inoculated with *S. oneidensis*, biofilm w/o enzyme: irradiation and 3 days inoculated with *S. oneidensis*.

SEM imaging provides evidence that surface area is one of, if not, the most important determinants in the potential for these polymers to biologically degrade (Figure 7 and Supporting Information Figures S19–S21). In the photodegradation step, the polymer undergoes superficial microcracking between the 0 h (i.e., no UV light) and 4 h UV light irradiated samples. This is in accordance with the microcracking that Li et al. reported, where an increase in the surface area was observed with an increase in photoaging time.^49^ Dramatic pitting and enlarged cracking was observed in the samples treated with enzyme. Interestingly, the enzyme pretreatment yields an image that suggests a different surface morphology on the irradiated plastic. While this could indicate UV degradation changes the way in which the enzyme degrades the surface (supported by Supporting Information Figure S19 with 2 h irradiated samples), it could also be an indication of a greater amount of the surface being degraded as evidenced by images that have lower magnifications (Supporting Information Figures S20 and S21). The samples that received both enzyme and biofilm growth show a further stage of degradation in comparison to that of the solely enzymatically treated samples, where we see enlarged cracking and additional roughening for the non-UV degraded samples. In the 4 h enzyme-bacteria sample, there is a decrease in the amount of pitting but there is an overall increase in the size of cracking and deeper pitting in comparison to the 0 h sample. Samples in which biofilms were grown on pristine plastic had substantial cracking and appeared similar to those in which biofilms were grown on enzymatically pretreated plastic. This indicates that the attached bacteria are effective at biodegrading the surface without and with enzyme pretreatment, although we know from biofilm quantification that it is to a lesser extent. Bacterial growth appears to be on or near the areas with the greatest amount of cracking (Supporting Information Figure S20; SEM images of same samples at lower magnification). There also seems to be a macroscale degradation pattern observed (Supporting Information Figure S21; lower magnification SEM images), where the UV light is likely providing some level of degradation or disruption to the orientation imparted on the polymer by the blown-film extrusion process. These degradation trends are similar to those observed in other enzymatic hydrolysis studies published regarding PLA.^50^ Overall, SEM imaging demonstrates the efficacy of the enzymatic pretreatment, as well as substantiating that biofilms also cause biodegradation of the polymer surface.

### Relating Results to Further Polymer Products

Further experiments were performed to determine if the trends observed in multistep biodegradation of PLA were consistent with other aliphatic polyesters, like PHBV, a potential alternative biodegradable polymer like PLLA, polycaprolactone (PCL) and polybutylene succinate (PBS) that are widely shown to break down in a variety of conditions including compost.^51^ First, we quantified enzymatic hydrolysis of the polymer as previously demonstrated with PLLA.^16^ PHBV showed no observable degradation by enzymes (Supporting Information Figures S22–S24). These results demonstrate that PHBV is rather recalcitrant to enzymatic hydrolysis, and further characterization of the polymer reveals an increase in crystallinity and little change to superficial chemistry (Supporting Information Figures S25–S27). These results contradict recent studies that have shown that PHBV is susceptible to enzymatic hydrolysis and biotic degradation,^52,53^ though this observation may be resulting from varied molecular weight, percentage of hydroxy-valerate, and differences in crystallinity of the starting polymer material as demonstrated with other biodegradable plastics. That is, previous work with PLA demonstrated that molecular weight, molecular weight distribution, and crystallinity may alter the rate of biodegradation.^54^ For PBS, high crystallinity can prohibit the permeation of water through the ester matrix preventing hydrolysis.^55^

**Figure 8:**
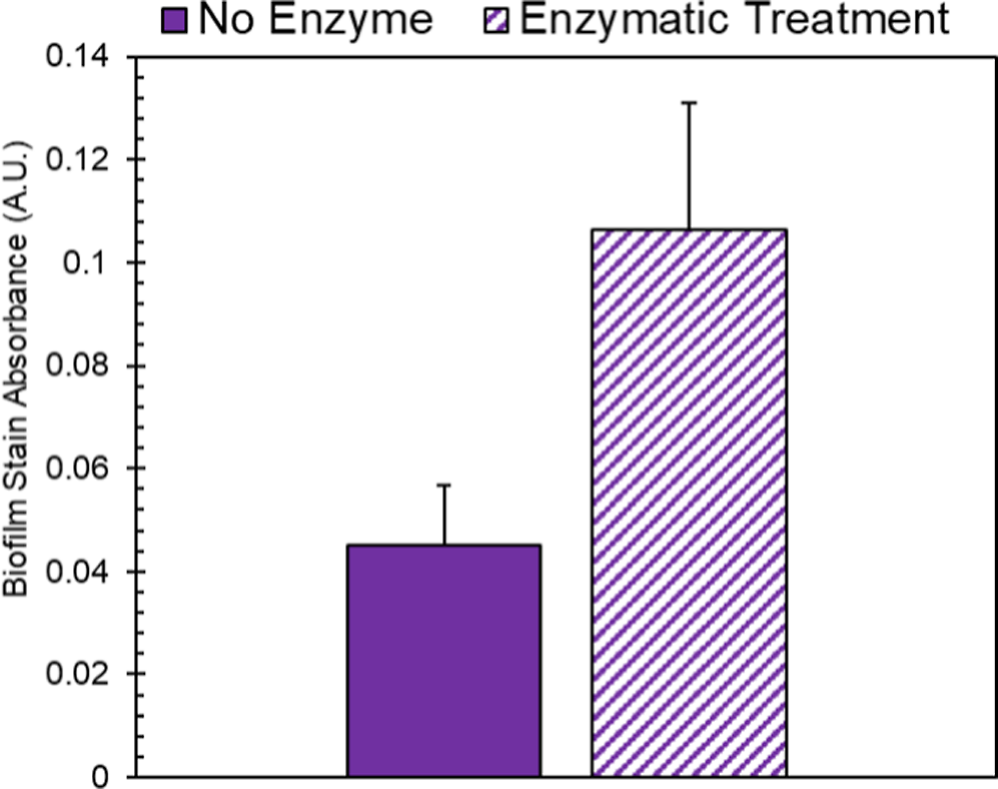
Bar graph showing biofilm growth on PHBV samples as quantified using crystal violet staining. Bars represent the average of samples (*n* = 3) with error representing the standard deviation. *t*-test between means reveals a significant different in the values (*p* = 0.0018).

We also explored if enzymatic pretreatment would be effective to promote biofilm formation. Figure 8 shows the biofilm growth on PHBV, and we observed that proK enzymatic pretreatment significantly (*p* < 0.05; *t*-test) promotes biofilm growth, even though overall growth of biofilm was one-tenth that of PLLA. In composting, the presence of microbial communities may provide a synergistic environment that facilitates the collective degradation of recalcitrant polymers.

## Conclusions

This study demonstrates that enzymes change the surface of biodegradable polymers to promote biofilm attachment, which could be an indicator of greater potential for biotic degradation. This relationship is seemingly driven by superficial roughening due to enzymatic hydrolysis as the chemistry of the surfaces is rather unchanging. Furthermore, photoaging plays an important role in the biotic degradation of PLLA. This relationship is likely due to the state of degradation where a combination of crystallinity, surface area, molecular weight, and accessible ester linkages prevents or promotes bacteria from attaching. The combination of these changing parameters results in nonlinear trends as it relates to the degree of photoaging, which may result in challenges predicting the fate of these materials upon environmental weathering. Evidence suggests that the biodegradability of a polymer is related to the amount of accessible surface area and the degradation state in which the polymer is in before the biological degradation process begins.

This work also shows that accelerated polymer biodegradation can be achieved through a multistep process, where there is a synergistic relationship between extracellular enzymes and the presence of a microbial community, like that found within a composting environment. Further applications of these systems demonstrated the ability to promote biotic degradation within other recalcitrant aliphatic polyesters. Additionally, this study demonstrates that a community system could be used in the remediation of biodegradable polymer build up within the environment. Consequently, this study serves to aid in identifying the ideal conditions for polymer biodegradation, which can further our ability to compost these polymers and inform the development, implementation, and end-of-life disposal of novel polymer systems.

## Supporting Information Available

Characteristics of the polymer films as received, method optimization and results of enzyme pretreatment, control tests with bovine serum albumin, results for characterization of EPS, additional planktonic cell and biofilm quantification, biofilm kinetic results, FTIR spectra and indices of degraded plastics, DSC thermograms and analyses of plastics, H NMR spectra and quantification, additional SEM images, methods, and results characterizing PHBV enzyme hydrolysis (PDF)
