# Supplementary material for: Effects of Enzyme Hydrolysis in Biofilm Formation and Biotic Degradation on Weathered Bioplastics
Source: ACS Omega. 2025 Apr 25;10(17):17394–403. doi: 10.1021/acsomega.4c10602 (PMC12059950; doi:10.1021/acsomega.4c10602)
Supplement: Supplementary file 1 — ao4c10602_si_001.pdf [file ao4c10602_si_001.pdf]

Supporting Information

Effects of Enzyme Hydrolysis in Biofilm Formation and Biotic Degradation on Weathered Bioplastics

Thomas D. Badzinski[1], Ariana L. Campanaro[2], Margaret H. Brown[1], Clare List,[1] R. Lee Penn[2], Melissa A. Maurer-Jones[1*]

1. Department of Chemistry and Biochemistry, University of Minnesota Duluth, Duluth MN 55812
2. Department of Chemistry, University of Minnesota, Minneapolis MN 55455

* - Corresponding author: maujones@d.umn.edu

Additional Plastic Sample Information

Table S1. Polymer properties of poly-L-lactic acid (PLLA) and poly(3-hydroxybutyrate-co-3-hydroxyvalerate) (PHBV) films as reported by the manufacturer. All polymers were purchased from Goodfellow USA (Huntingdon, UK).

Characteristic	PLLA	PHBV
Density (g/cm^3)	1.25	1.25
Thickness/diameter (mm)	0.05	0.01
Orientation	Biaxial	Biaxial
Molecular Weight (g/mol)	Not reported	Not reported
Morphology	Film	Film
Elongation at Break (%)	6	15
Tensile Strength (MPa)	53	28

Determining parameters of enzyme pre-treatment

To determine the optimum conditions for the enzymatic pre-treatment of the PLLA samples, several experiments were performed to see if concentration or time of enzyme hydrolysis influences biofilm formation. Seen in Figure S1A, samples were soaked in 10 mL Tris-HCl buffer (50 mM; pH 7.5), incubated at 30°C, and received varying 1 mL doses (0-3) of 90 µM proteinase K (proK, RPI) at 12 h intervals. Resulting proK concentrations were 0 µM, 8.2 µM, 15 µM, and 20.8 µM. After, samples were transferred to the biofilm growth assay. In Figure S1B, samples were soaked in 10 mL Tris-HCl buffer (50 mM; pH 7.5), incubated at 30°C, and received one 1-mL dose of 90 µM proK (final solution concentration of 8.2 µM) at varying time intervals leading to exposure times of 12 h, 24 h, and 36 h. After, samples were transferred to the biofilm growth assay. Figure S1A shows a significant increase ($p<0.05$; t-test) in stain absorbance with the addition of one dose of enzyme. Further, a significant decrease in stain absorbance was observed beyond one dose. This helped establish that one dose of enzyme was optimal for biofilm growth volume. In Figure S1B, there is a significant decrease in stain absorbance between 36 h and 24 h of enzymatic exposure time, but no statistical difference was observed between 36 h and 12 h. This helped establish there was no statistical benefit to pre-treating the polymer surface for longer than 12 h.

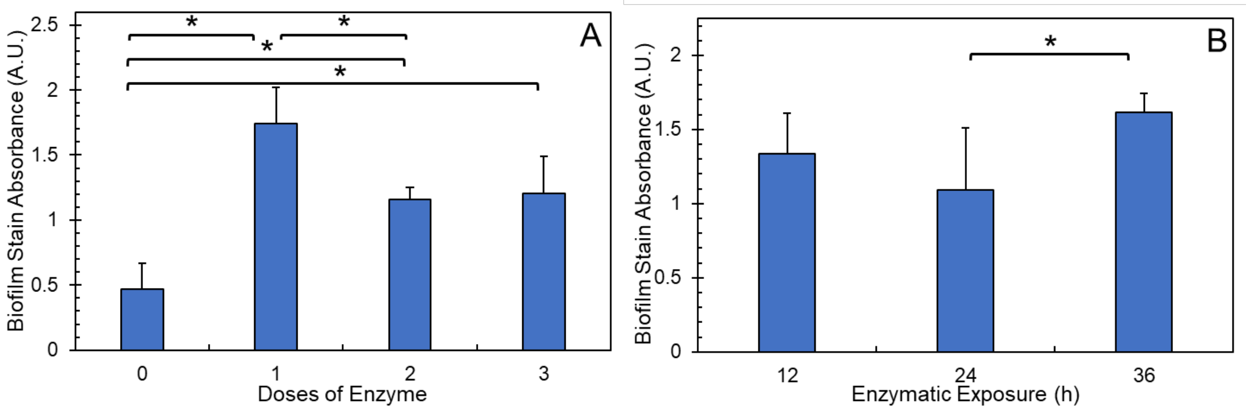

Figure S1. A). Biofilm growth on 0 h PLA with varying doses of proK. Polymer time spent in solution was 36 h for all samples. Samples were done in triplicate and averaged with error bars being standard deviation. B). Biofilm growth with varying amounts of time exposed to one dose of proK. Polymer time spent in solution was 36 h for all samples. Bars are the average of samples (n=5) with the error bars representing the standard deviation. * $P<0.05$ from t-test.

Pretreatment of Plastics with Bovine Serum Albumin (BSA)

To assess whether irreversible protein binding was acting as a scaffolding for the biofilm to form on, a biofilm growth assay was performed with a bovine serum albumin (BSA) pre-treatment replacing the enzymatic pre-treatment. Samples were soaked in 10 mL Tris-HCl buffer (50 mM; pH 7.5) and received varying 1 mL doses (0-3) of 90 µM BSA (EMD Millipore) at 12 h intervals. Resulting concentrations were 0 µM, 8.2 µM, 15 µM, and 20.8 µM. After, samples were rinsed with methanol and transferred to the biofilm growth assay. Seen in Figure S2, no trend is observed, and no statistical difference was observed between 0 doses and 1-3 doses as it relates to the amount of biofilm formed. This helped establish that the bacteria were not using irreversible protein binding, between the polymer substrate and the enzyme, as a nucleation site for biofilm growth.

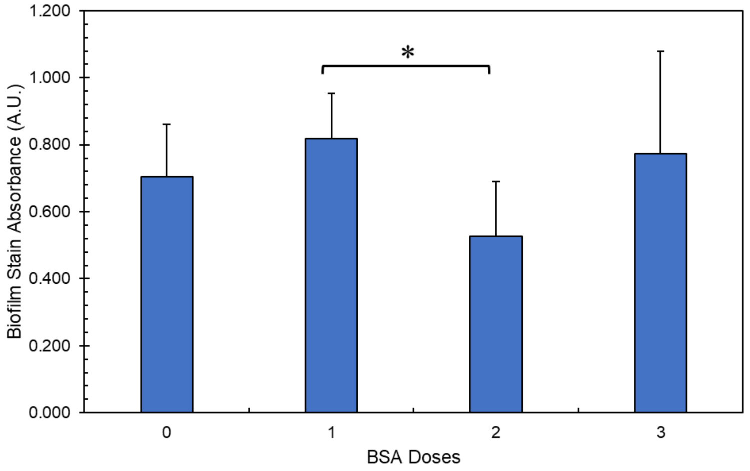

Figure S2. Biofilm growth on 0h PLA exposed to varying doses of BSA. Polymer time spent in solution was 36 h for all samples. Bars represent the average (n=4) of the samples with the error bars representing standard deviation. * $P<0.05$ from t-test.

Characterization of Extracellular Polymeric Substance (EPS)

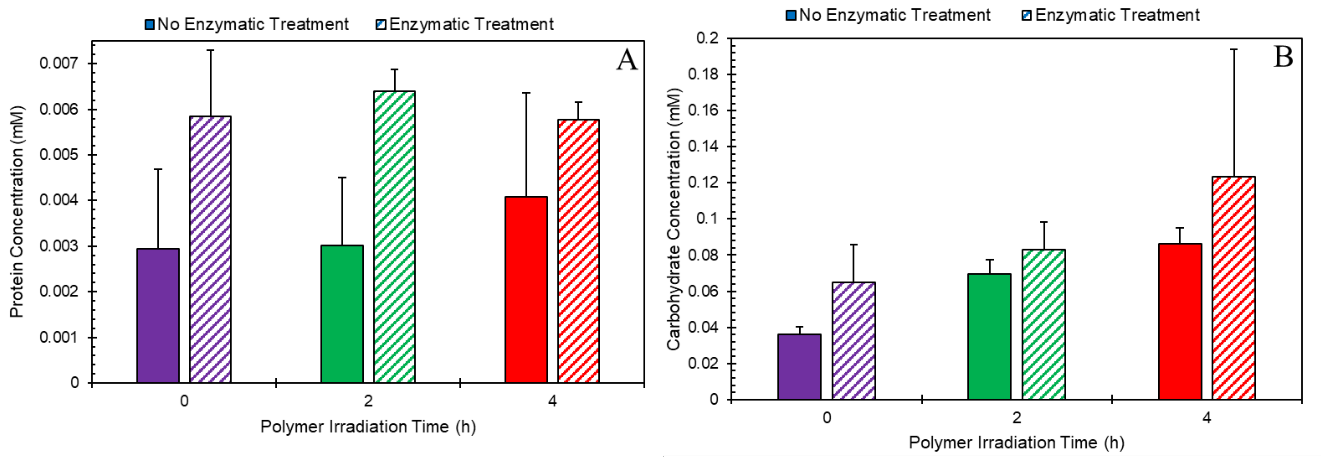

Figure S3. Characterization of the Extracellular Polymeric Substance (EPS) components of *S.oneidensis* biofilm growth. A). Protein concentrations in the EPS as measured by UV-Vis (λ=500 nm) with Lowry's assay, B). Carbohydrate concentration in EPS as measured by UV-Vis (λ=480 nm) after phenol-sulfuric acid assay. Samples were taken in triplicate and blank subtracted; error bars represent standard deviation of measurements.

Planktonic Cells Remaining after Biofilm Growth and Buffer Soak

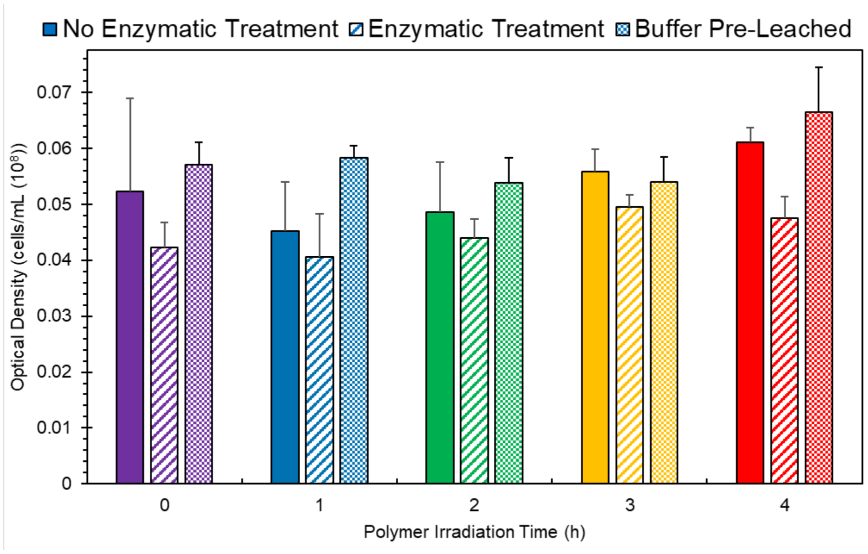

Figure S4. Optical density at 600 nm of remaining M4 nutrient broth after 3 days of biofilm growth. Before biofilm growth, samples were control group (solid - no pretreatment), enzymatically degraded (striped), or soaked in Tris-HCl buffer (checkered). Bars represent the average (n=3) of the samples with error bars representing standard deviation.

Biofilm Growth on Polymers Soaked in Buffer

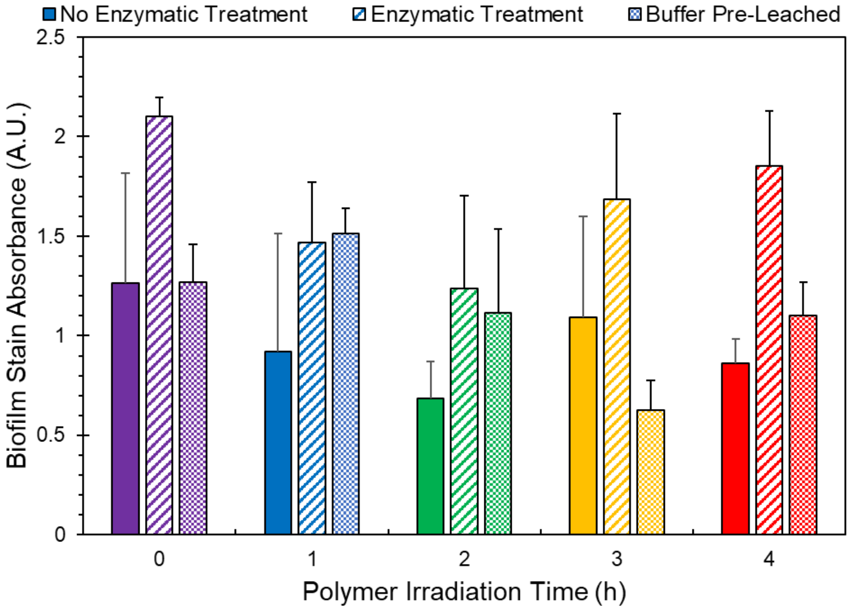

Figure S5. Biofilm abundance as measured by the uptake of crystal violet stain after 3 days of biofilm growth. Before biofilm growth, samples were control group (solid - no pretreatment), enzymatically degraded (striped), or soaked in Tris-HCl buffer (checkered). Bars represent the average (n=3) of the samples with error bars representing standard deviation.

Tracking Biofilm Growth Kinetics

Following the kinetics of biofilm formation over 7 days reveals important trends as related to the weathering of the polymer (Figure 3). Within the 0 h PLLA sample, there was an increase of the amount of biofilm through the first three days that slowly declined across the remaining four days. This may be indicative that availability of consumable nutrients declined or that there was a bacteria saturation on the polymer surface, causing intraspecies competition for nutrients and die-off. In the 4 h PLLA samples, the first three days show less growth in comparison to the 0 h sample, but the biofilm growth accelerates the remaining four days, peaking at day 7. This may be due to the release of available nutrients in the form of monomeric, dimeric, or oligomeric lactide, providing food for the bacteria that does not require a biofilm to form until the readily available nutrients were consumed (past day 3). Whereas the 0 h sample bacteria show fast growth to form biofilm, which is required for survival, but may die out over time due to an inability to degrade pristine polymer or other nutrient limitations. This trend in biofilm kinetics, as it relates to the photo-degradation state of the plastic, may suggest that the biofilm is being supported by bacterially degraded, digestible products, where the 0 h sample may be unable to sustain the growth levels in comparison to the 4 h PLLA. This conclusion is supported by the increase in carbohydrates observed in the EPS analysis, where protein content stays constant, but the carbohydrate content increases relative to the degradation state.

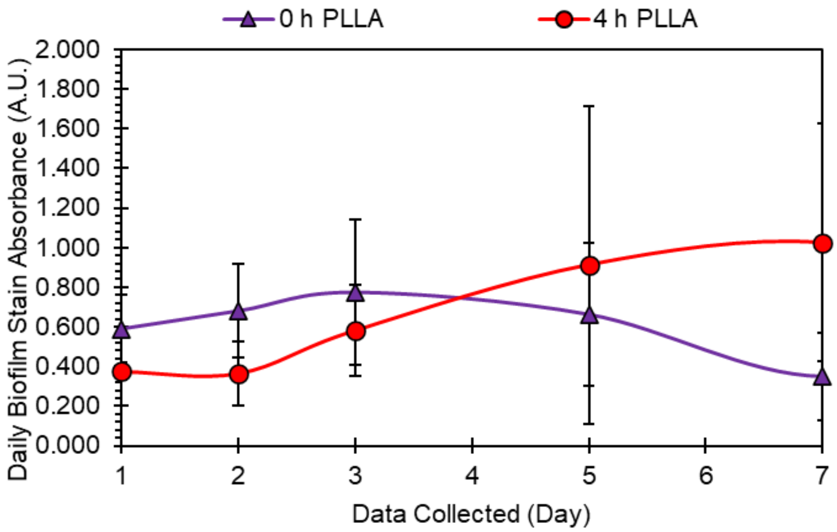

Figure S6. *S.oneidensis* biofilm growth over 7 days as measured through crystal violet staining. Samples did not receive any enzymatic pre-treatment. Markers represent the average (n=3) with the error bars the standard deviation.

FTIR spectra of samples

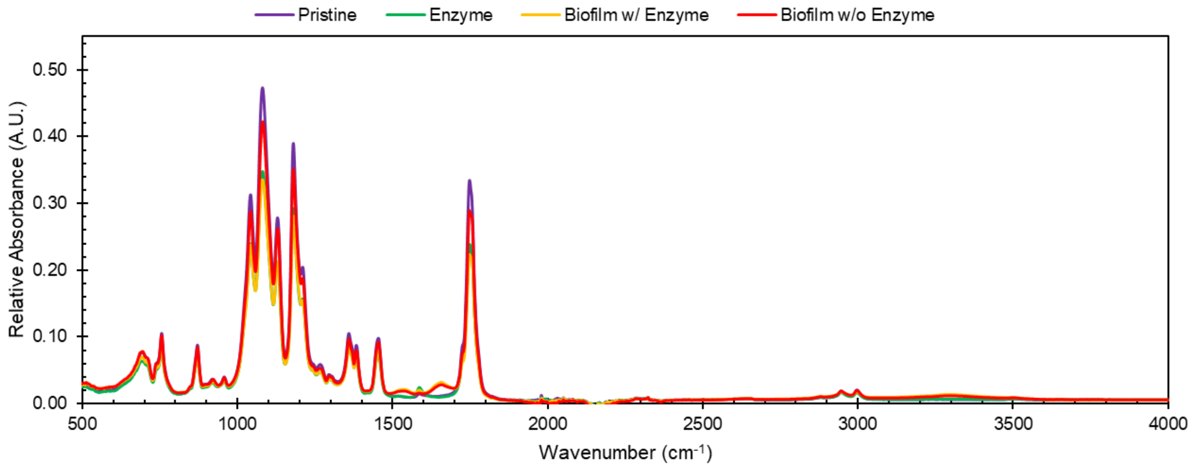

Figure S7. Averaged 0 h PLA FTIR spectra grouped by polymer irradiation time. Pristine/UV - Irradiation, Enzyme - Irradiation & 12 h in 8.2 µM ProK, Biofilm with (w/) Enzyme - Irradiation, 12 h in 8.2 µM ProK, & 3 days inoculated with *S.oneidensis*, Biofilm without (w/o) Enzyme - Irradiation & 3 days inoculated with *S.oneidensis*. Samples ran in triplicate and averaged.

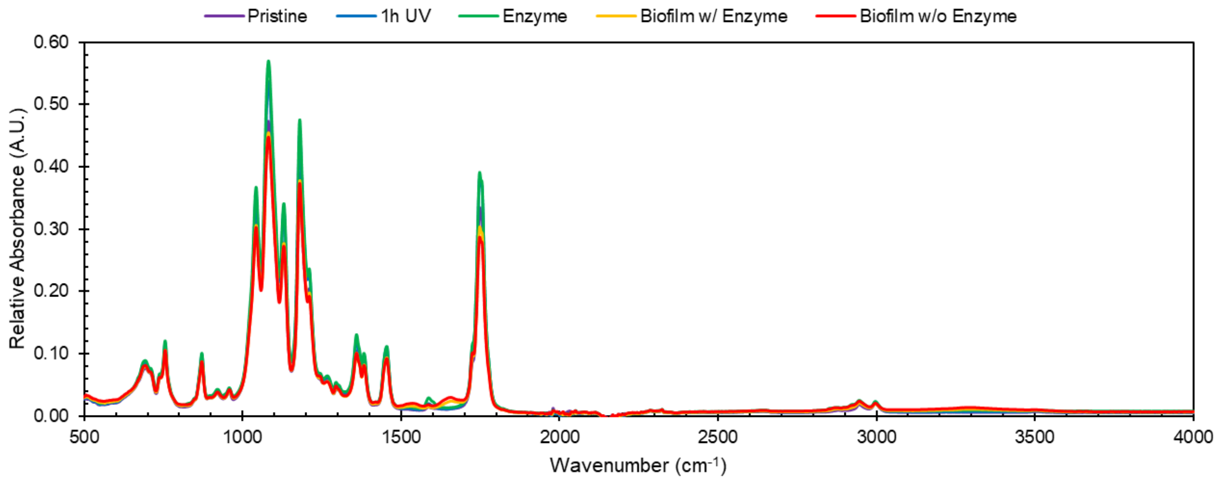

Figure S8. Averaged 1 h PLA FTIR spectra grouped by polymer irradiation time. Pristine/UV - Irradiation, Enzyme - Irradiation & 12 h in 8.2 µM ProK, Biofilm with (w/) Enzyme - Irradiation, 12 h in 8.2 µM ProK, & 3 days inoculated with *S.oneidensis*, Biofilm without (w/o) Enzyme - Irradiation & 3 days inoculated with *S.oneidensis*. Samples ran in triplicate and averaged.

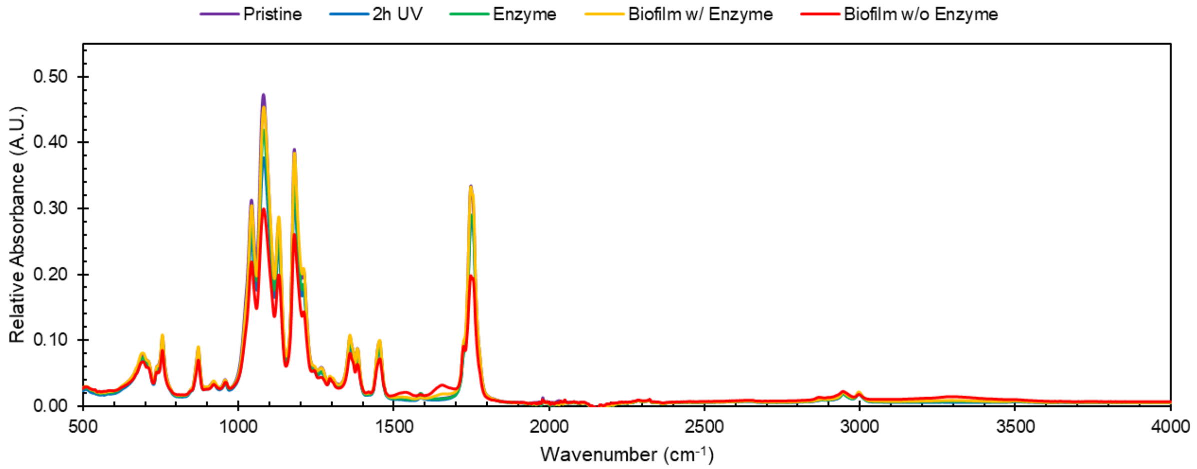

Figure S9. Averaged 2 h PLA FTIR spectra grouped by polymer irradiation time. Pristine/UV - Irradiation, Enzyme - Irradiation & 12 h in 8.2 µM ProK, Biofilm with (w/) Enzyme - Irradiation, 12 h in 8.2 µM ProK, & 3 days inoculated with *S.oneidensis*, Biofilm without (w/o) Enzyme - Irradiation & 3 days inoculated with *S.oneidensis*. Samples ran in triplicate and averaged.

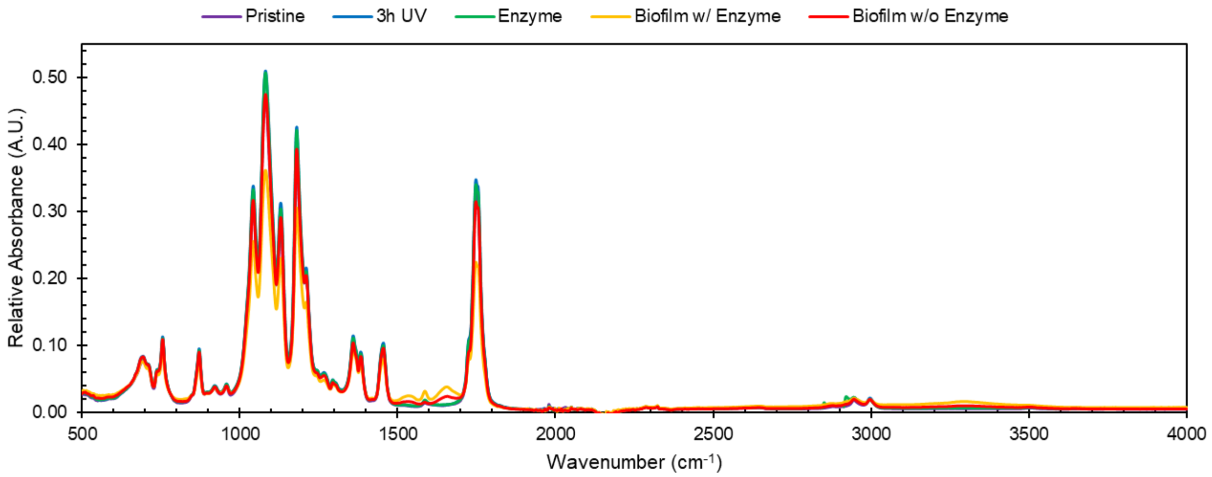

Figure S10. Averaged 3 h PLA FTIR spectra grouped by polymer irradiation time. Pristine/UV - Irradiation, Enzyme - Irradiation & 12 h in 8.2 µM ProK, Biofilm with (w/) Enzyme - Irradiation, 12 h in 8.2 µM ProK, & 3 days inoculated with *S.oneidensis*, Biofilm without (w/o) Enzyme - Irradiation & 3 days inoculated with *S.oneidensis*. Samples ran in triplicate and averaged.

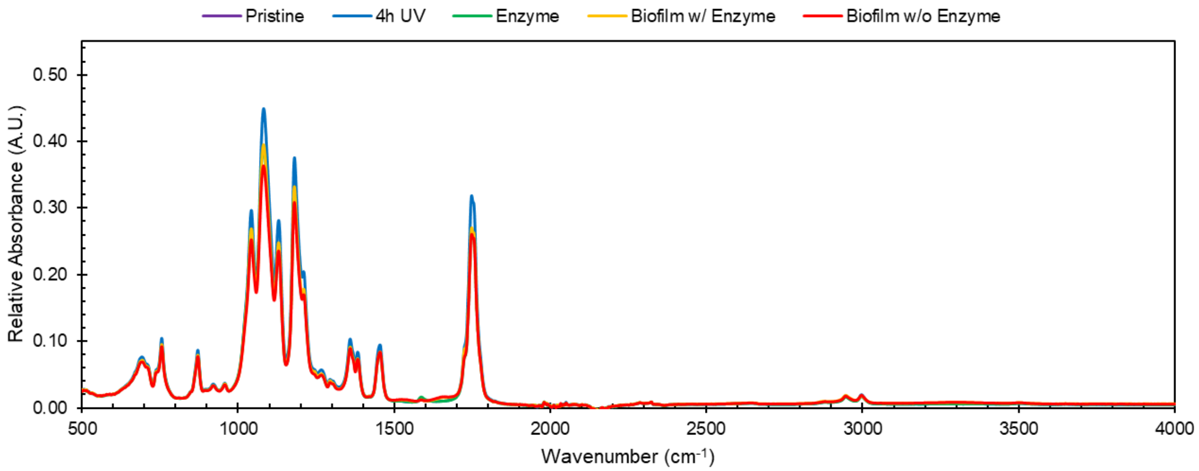

Figure S11. Averaged 4 h PLA FTIR spectra grouped by polymer irradiation time. Pristine/UV - Irradiation, Enzyme - Irradiation & 12 h in 8.2 µM ProK, Biofilm with (w/) Enzyme - Irradiation, 12 h in 8.2 µM ProK, & 3 days inoculated with *S.oneidensis*, Biofilm without (w/o) Enzyme - Irradiation & 3 days inoculated with *S.oneidensis*. Samples ran in triplicate and averaged.

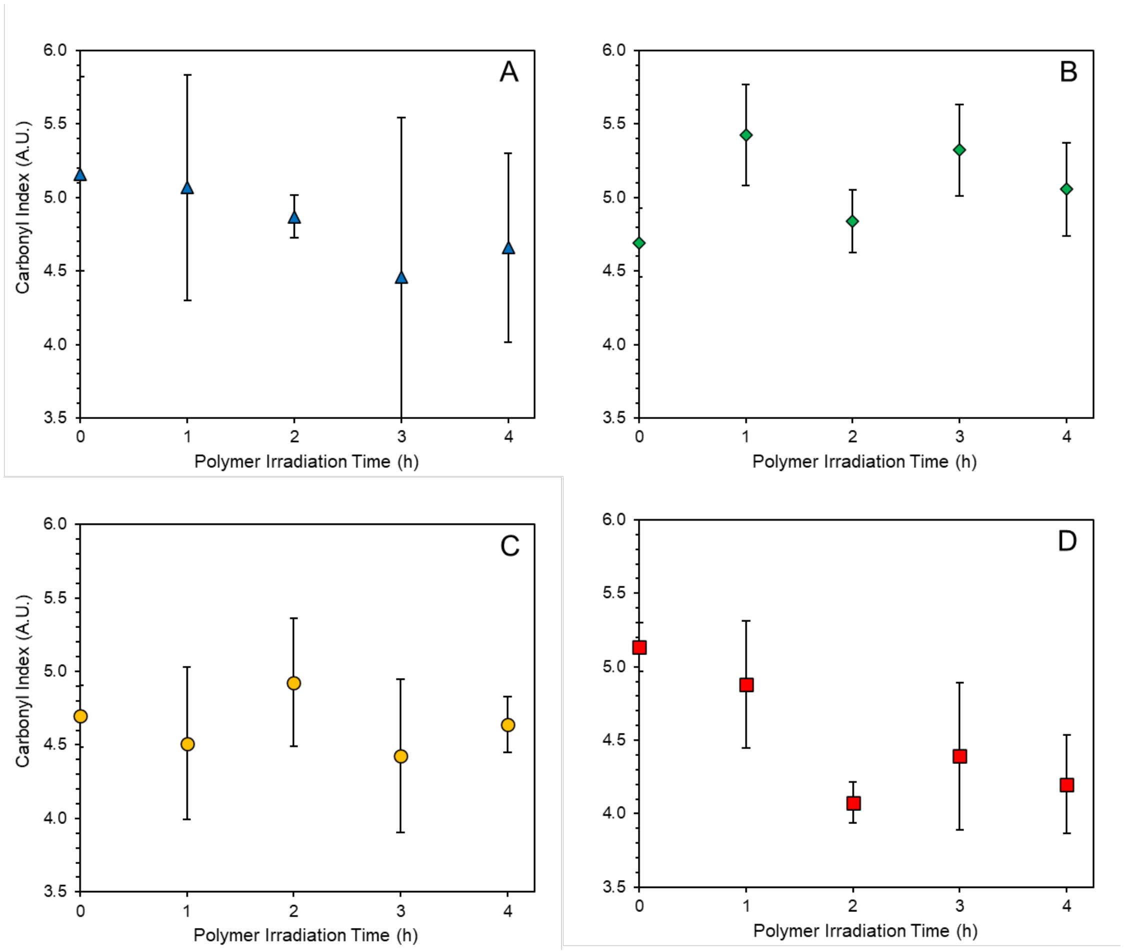

Figure S12. Carbonyl index (1710-1810 cm^{-1}/1455 cm^{-1}) of samples receiving A.) UV - Irradiation, B.) Enzyme - Irradiation & 12h in 8.2 μM ProK, C.) Biofilm with (w/) Enzyme - Irradiation, 12h in 8.2 μM ProK, & 3 days inoculated with *S.oneidensis*, D.) Biofilm without (w/o) Enzyme - Irradiation & 3 days inoculated with *S.oneidensis*. Markers represent the average of samples (n=3) with error bars representing the standard deviation.

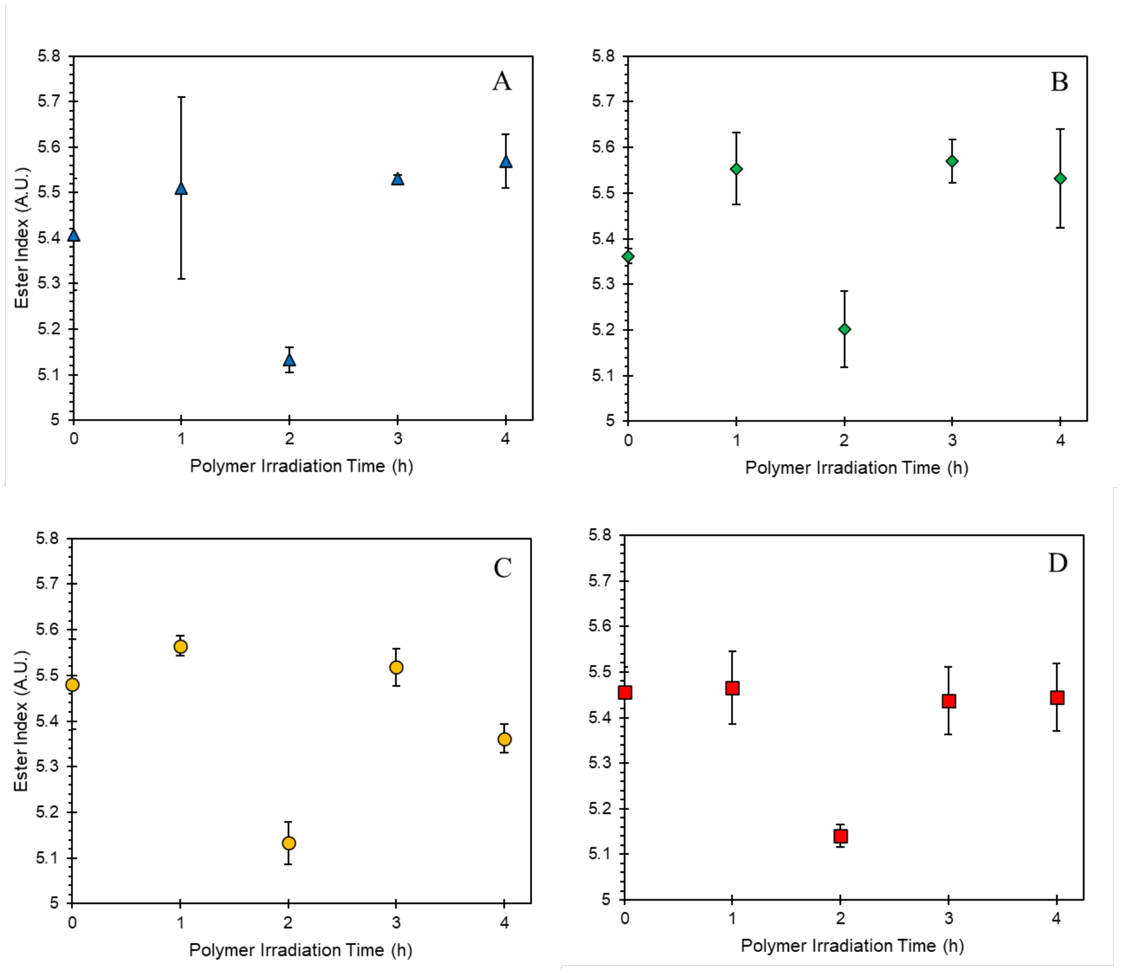

Figure S13. Ester index (1150-1250 cm^{-1}/1455 cm^{-1}) of samples receiving A.) UV - Irradiation, B.) Enzyme - Irradiation & 12h in 8.2 µM ProK, C.) Biofilm with (w/) Enzyme - Irradiation, 12h in 8.2 µM ProK, & 3 days inoculated with *S.oneidensis*, D.) Biofilm without (w/o) Enzyme - Irradiation & 3 days inoculated with *S.oneidensis*. Markers represent the average of samples (n=3) with error bars representing the standard deviation.

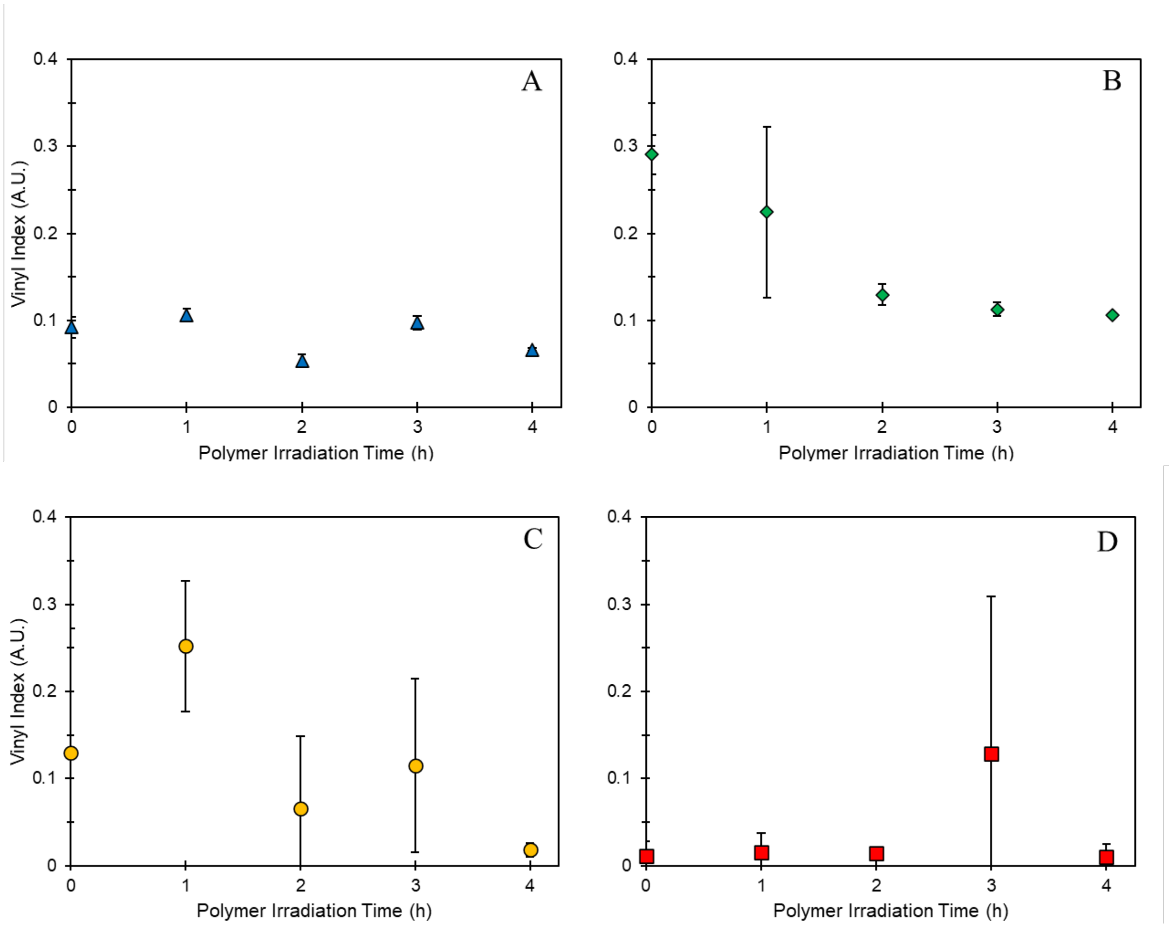

Figure S14. Vinyl index (1600-1700 cm^{-1}/1455 cm^{-1}) of samples receiving A.) UV - Irradiation, B.) Enzyme - Irradiation & 12h in 8.2 µM ProK, C.) Biofilm with (w/) Enzyme - Irradiation, 12h in 8.2 µM ProK, & 3 days inoculated with *S.oneidensis*, D.) Biofilm without (w/o) Enzyme - Irradiation & 3 days inoculated with *S.oneidensis*. Markers represent the average of samples (n=3) with error bars representing the standard deviation.

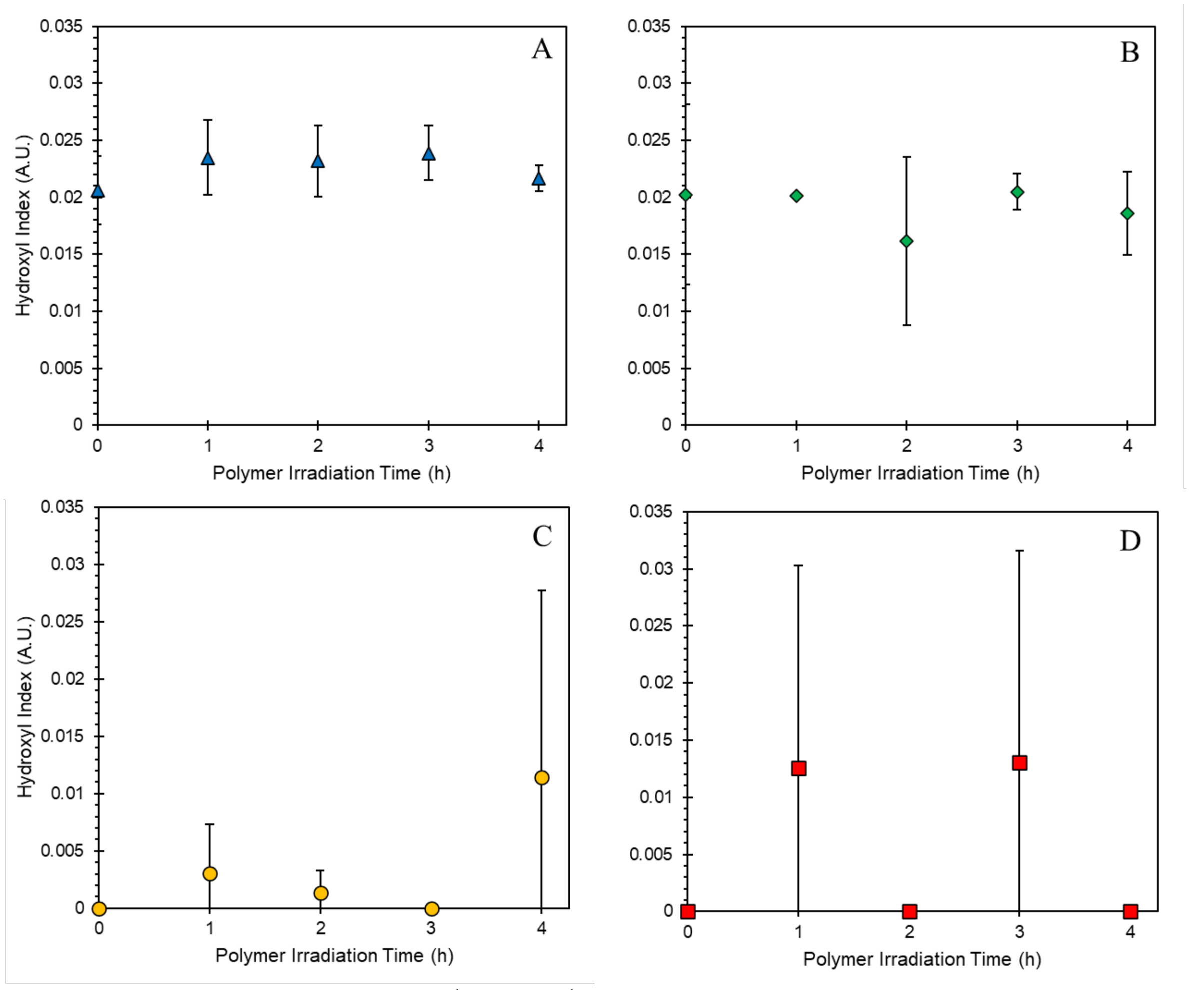

Figure S15. Hydroxyl index (3000-3600 cm^{-1}/1455 cm^{-1}) of samples receiving A.) UV - Irradiation, B.) Enzyme - Irradiation & 12h in 8.2 µM ProK, C.) Biofilm with (w/) Enzyme - Irradiation, 12h in 8.2 µM ProK, & 3 days inoculated with *S.oneidensis*, D.) Biofilm without (w/o) Enzyme - Irradiation & 3 days inoculated with *S.oneidensis*. Markers represent the average of samples (n=3) with error bars representing the standard deviation.

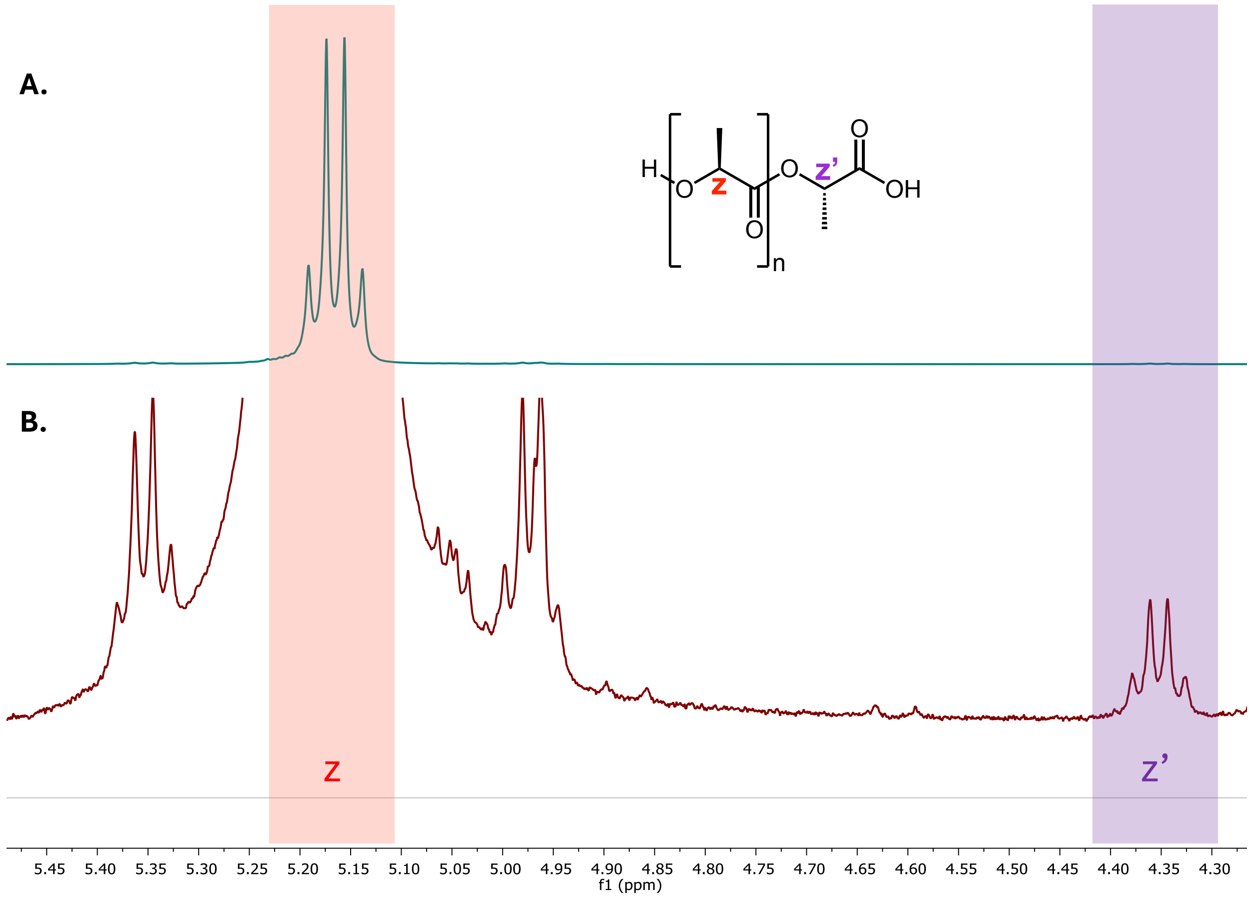

Figure S16. NMR spectra of 0h pristine PLA demonstrating the peaks of the of the methine protons, with B showing the peaks closer to the baseline of A in order to observe the end member methine proton (z').

Table S2: ^{1}H NMR of samples before and after biofilm formation (400 Mz; CDCl3; 16 scans; both bands are quadruplicates; $\delta_{end\ group}$=4.35 ppm; $\delta_{internal}$ =5.17 ppm)

	**UV Degradation**		**Post Biofilm with Enzyme Pretreatment**		**Post Biofilm without Enzyme**	
UV light Time	*Ratio of integrates of Internal/End Group Methine Proton*	*Conversion to M_n**	*Ratio of integrates of Internal/End Group Methine Proton*	*Conversion to M_n**	*Ratio of integrates of Internal/End Group Methine Proton*	*Conversion to M_n**
0 h (pristine)	262	18,900	226	16,300	229	16,600
2 h UV light	229	16,600	211	15,300	202	14,600
4 h UV light	208	15,050	200	14,500	224^{α}	$16{,}200^{\alpha}$

* Conversion to M_n as described in Pérez et. al., with the ratio of integrals the provides a repeating unit value of n.[3]

$^{\alpha}$ Small sample size contributed to a low signal to noise with this sample, which likely contributed to error within the integration. However, to be consistent, fit parameters were kept the same for all ^{1}H NMR sampling

Additional DSC Analysis

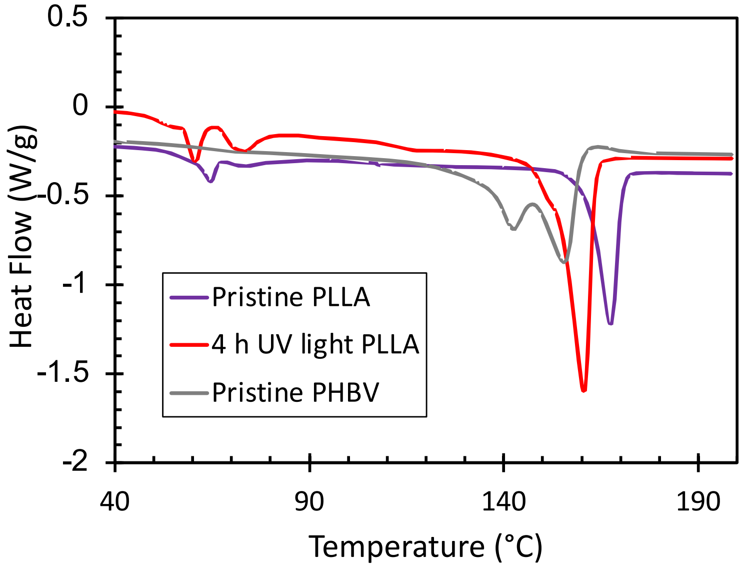

Figure S17. DSC thermograms of the first heat cycle for polymer samples.

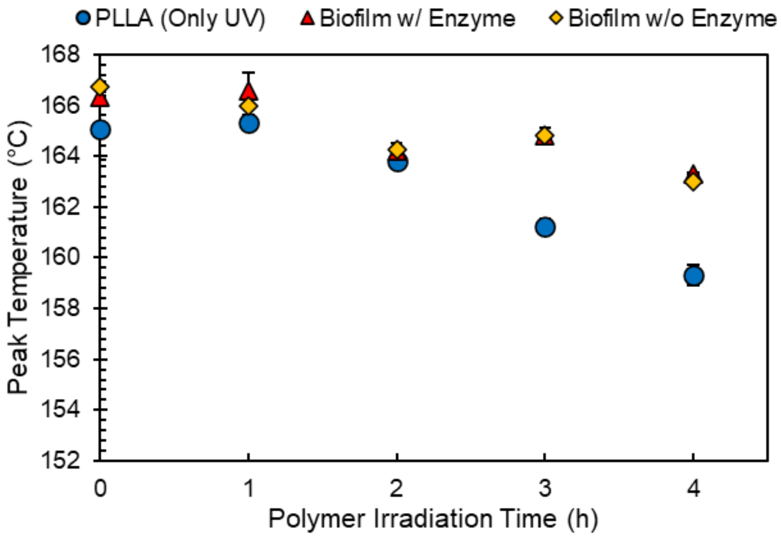

Figure S18. Peak temperature of samples upon various UV light and biological treatments as measured with DSC. Peak temperature related to the molecular weight of the samples. Markers represent the average of samples (n=3) with error bars representing standard deviation.

Additional Scanning Electron Microscopy (SEM) Images with Varied Magnficiations

Magnification the same as shown in manuscript Figure 7.

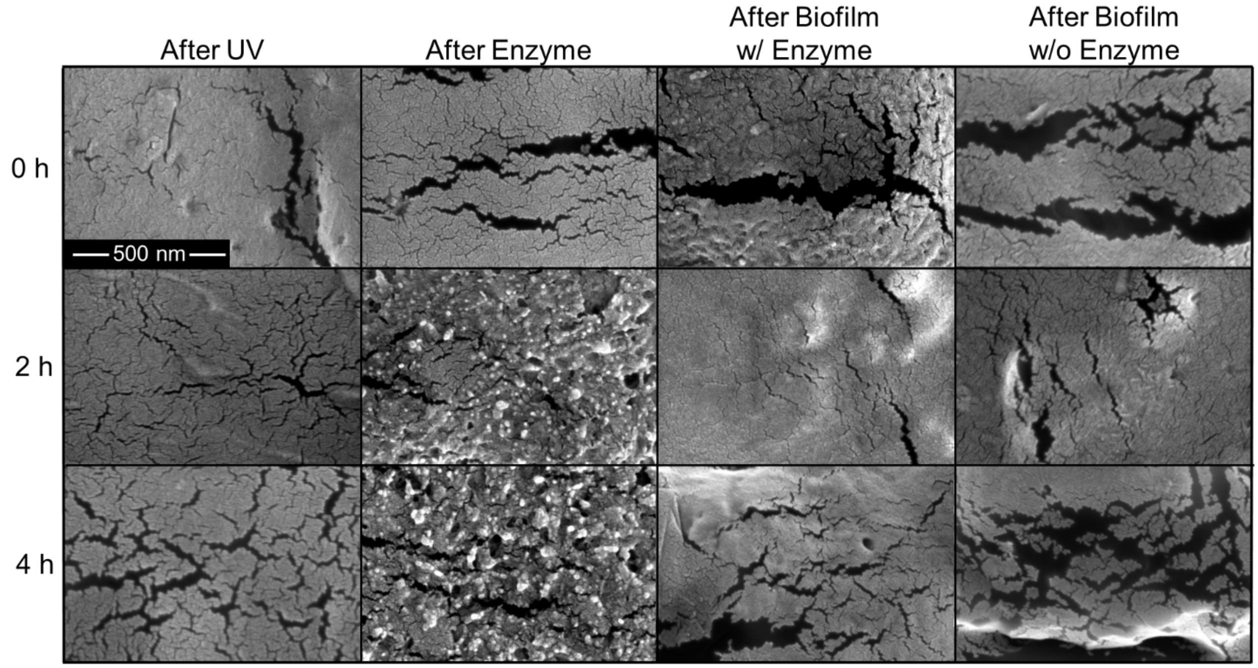

Figure S19. SEM images of PLLA with no, 2 h or 4 h UV light irradiation and exposed to varying biological degradation treatments. Conditions are as follows, UV - Irradiation, After Enzyme - Irradiation & 12h in 90 µM ProK, After Biofilm with (w/) Enzyme - Irradiation, 12h in 90 µM ProK, & 3 days inoculated with *S.oneidensis*. After Biofilm without (w/o) Enzyme - Irradiation, & 3 days inoculated with *S.oneidensis*.

Decreased magnification of SEM Images with 1-3 μm scale bars.

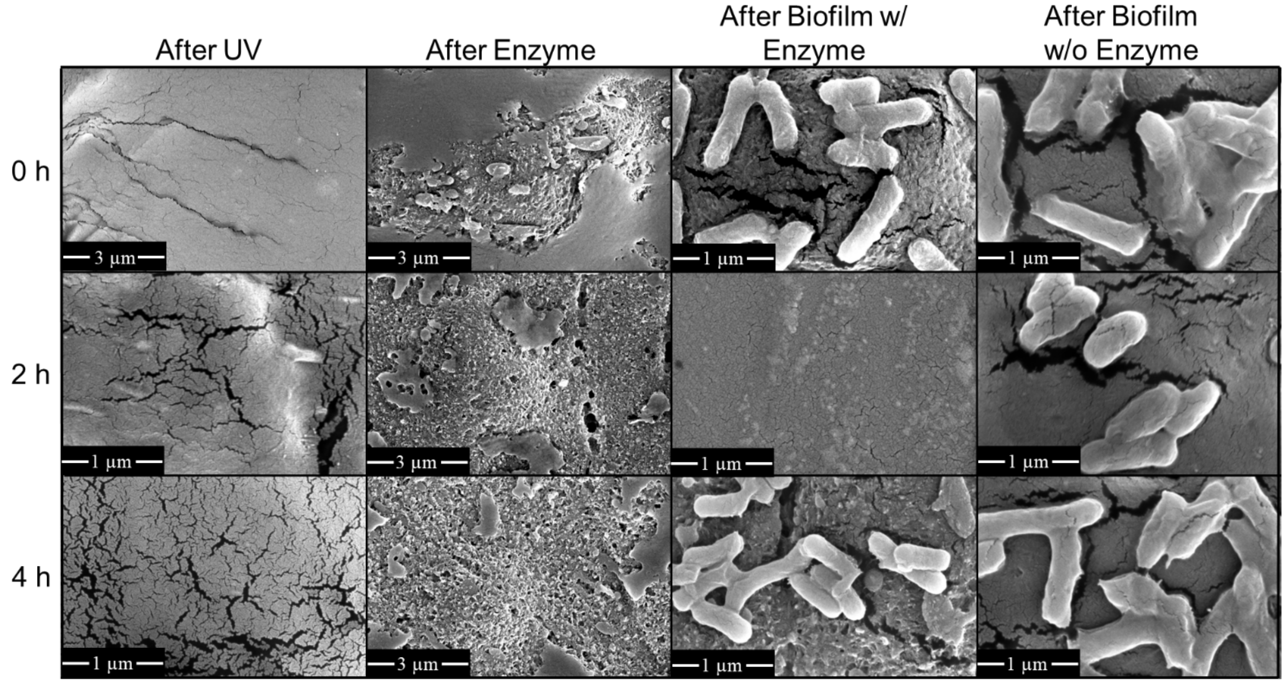

Figure S20. SEM images of PLLA with no, 2 h or 4 h UV light irradiation and exposed to varying biological degradation treatments. Conditions are as follows, UV - Irradiation, After Enzyme - Irradiation & 12h in 90 µM ProK, After Biofilm with (w/) Enzyme - Irradiation, 12h in 90 µM ProK, & 3 days inoculated with *S.oneidensis*. After Biofilm without (w/o) Enzyme - Irradiation, & 3 days inoculated with *S.oneidensis*.

Decreased magnification of SEM Images with 100-300 μm scale bars.

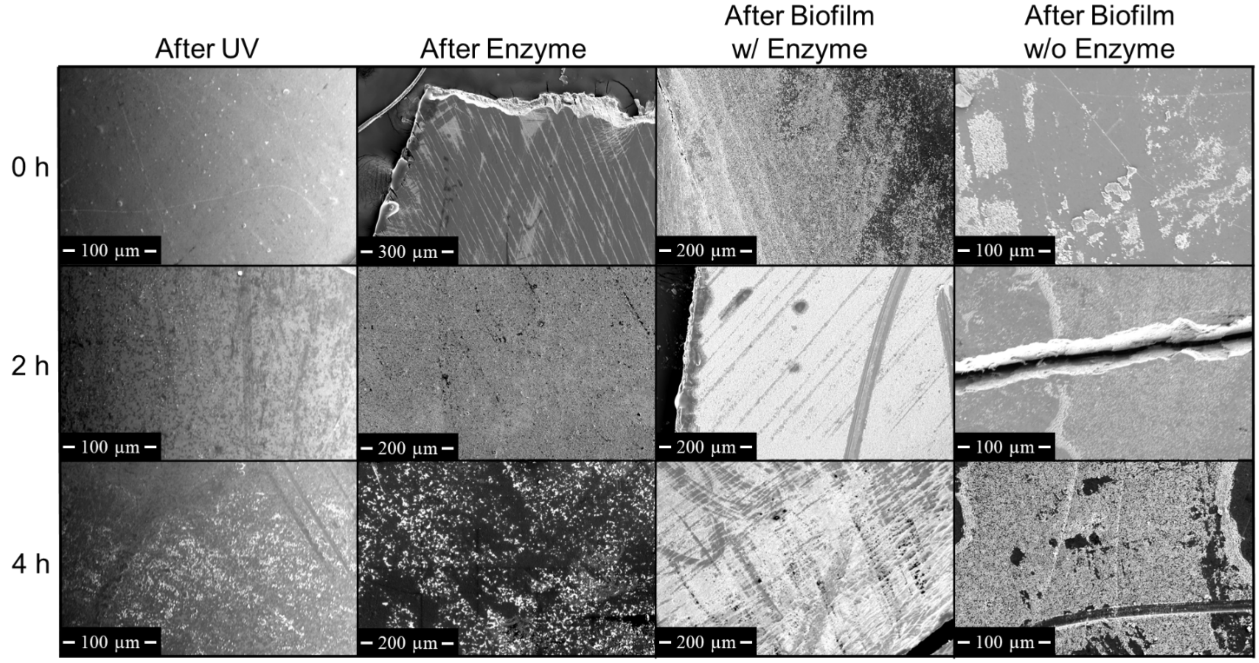

Figure S21. SEM images of PLLA with no, 2 h or 4 h UV light irradiation and exposed to varying biological degradation treatments. Conditions are as follows, UV - Irradiation, After Enzyme - Irradiation & 12h in 90 μM ProK, After Biofilm with (w/) Enzyme - Irradiation, 12h in 90 μM ProK, & 3 days inoculated with *S.oneidensis*. After Biofilm without (w/o) Enzyme - Irradiation, & 3 days inoculated with *S.oneidensis*.

Methods for Quantifying Enzymatic Degradation of PHBV with Lipase and ProK

Lipase Preparation

Lipase was purchased from Fisher Scientific as Fisher Science Education™ Lipase Powder (CAS: 9001-62-1). Solutions were prepared in 0.01M Tris-HCl buffer at pH 8. Due to contamination of the purchased lipase, solutions were purified with gravity filtration using a 9 cm, P5 grade filter with a 5 to 10 µm particle retention rating. UV-VIS spectrometer (Cary-5000 UV-Visible/Near IR double beam spectrometer) was used to confirm and/or correct the lipase concentration after the purification step. Analysis of absorbance at 280 nm allowed for the determination of the concentration of lipase standard using Beer's law ($A = \varepsilon bC$) and a previously reported molar absorptivity value of 37,000 $M^{-1}cm^{-1}$.[1] Standards were prepared to an initial concentration of 21 µM, and the final concentration was determined prior to data analysis.

Fluorescence Spectroscopy of Enzymatic Hydrolysis

Quantification of the enzymatic hydrolysis of polymers was adopted from previous studies (Brown et al., 2023). The initial step in the preparation of the biopolymer samples was to add a fluorogenic probe (fluorescein dilaurate - FDL) to the photoirradated polymer. This was performed by adding the fluorogenic probe to a solution of the polymer dissolved in chloroform. From here, the samples were cast in quartz fluorescence cuvettes, where the samples formed a small film that conformed to the bottom of the cuvette. These samples were then placed in a Horiba FluoroMax-4 Spectrofluorometer, and the enzyme solution (30 µM concentration) was added. Data was collected every 5 minutes, over roughly eight hours. Data was then analyzed using an FDL calibration curve and the percentage of polyester bonds broken was able to be determined.[2]

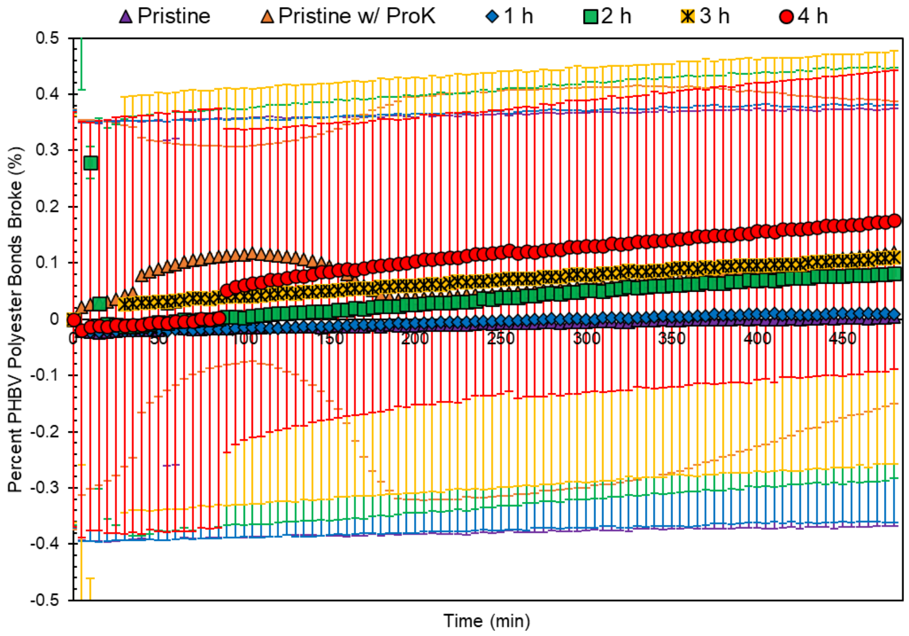

Figure S22. Percentage of polyester bonds broken over time using lipase, unless otherwise specified, of variously UV aged PHBV. Markers represent the average of samples (n=3) and the error bars represent standard deviation.

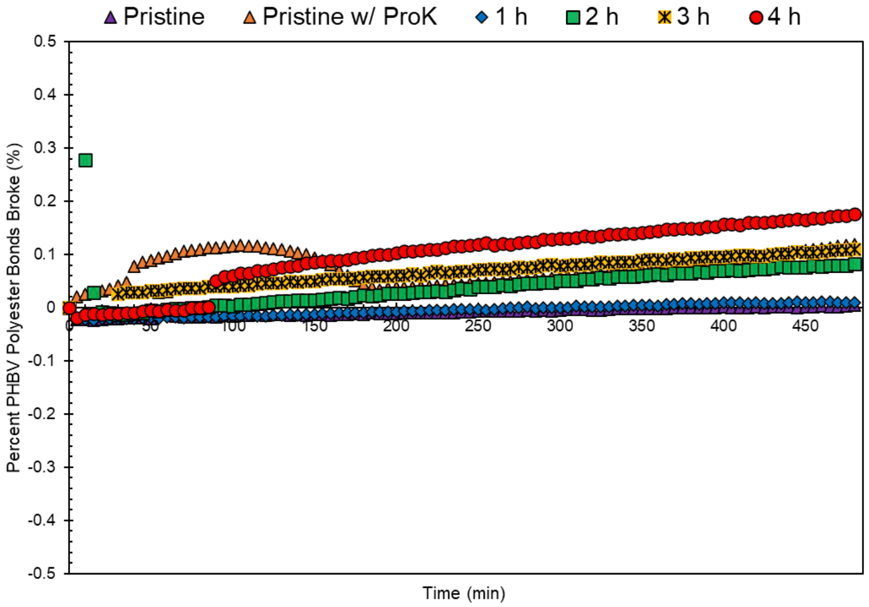

Figure S23. Percentage of polyester bonds broken over time using lipase, unless otherwise specified, of variously UV aged PHBV. Error bars removed for clarity.

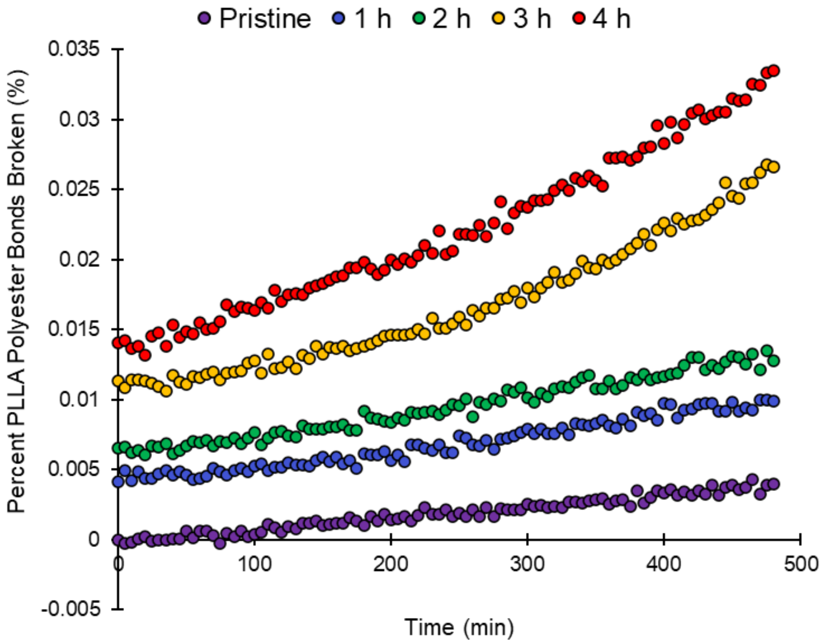

Figure S24. Percentage of polyester bonds broken over time of the enzyme hydrolysis experiments with samples only exposed to buffer.

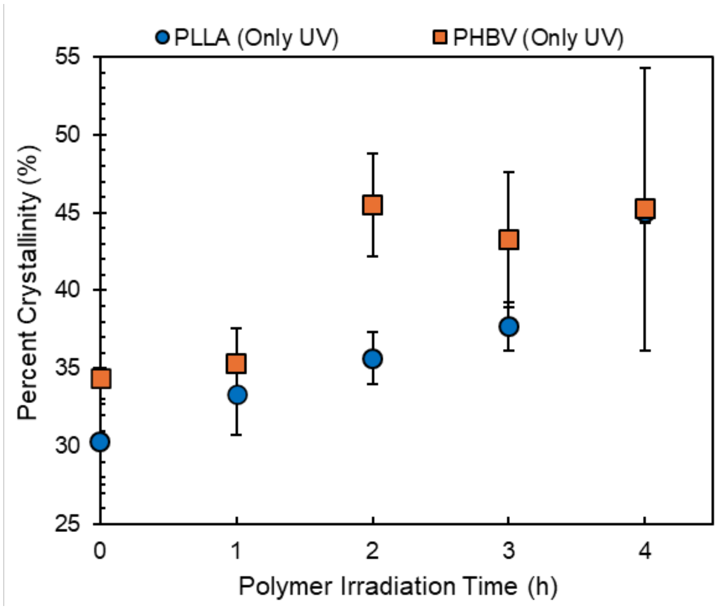

Figure S25. Percent crystallinity (%) of irradiated PLLA and PHBV as determined by DSC. Markers represent the average of samples (n=3) with error bars representing standard deviation.

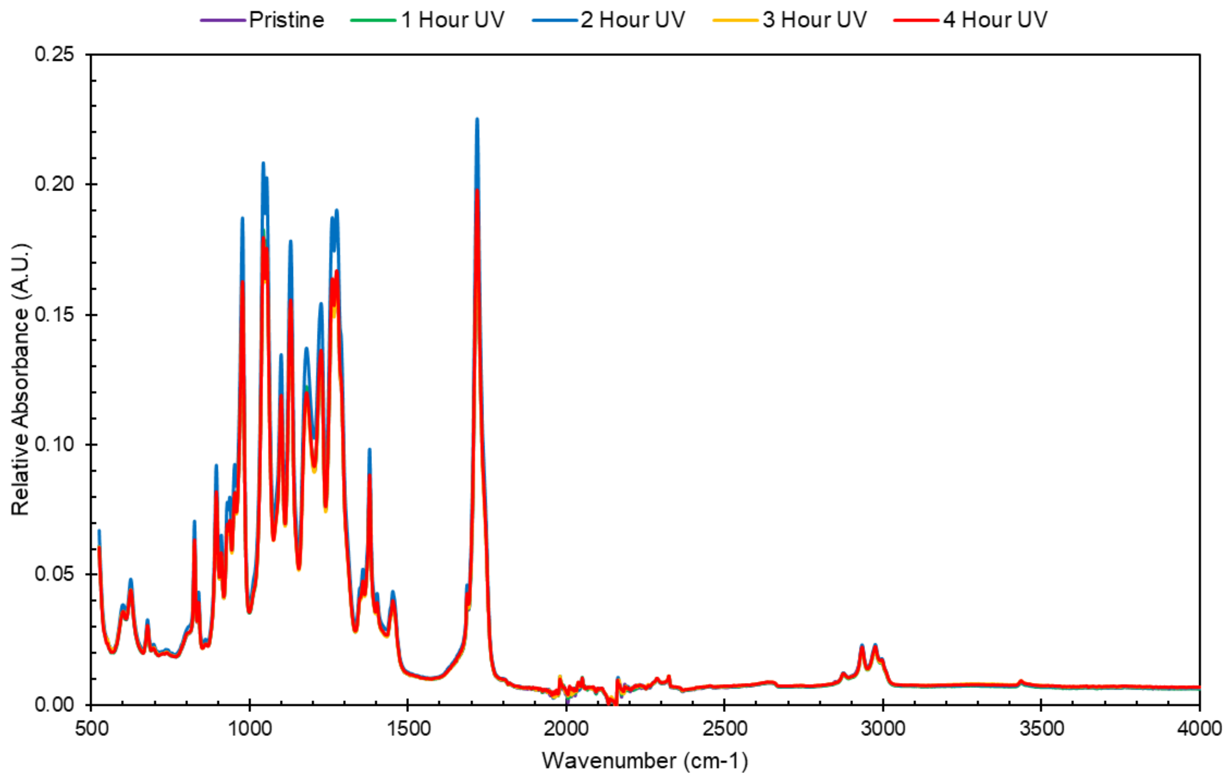

Figure S26. Averaged PHBV FTIR spectra by polymer irradiation time. Samples ran in duplicate and averaged.

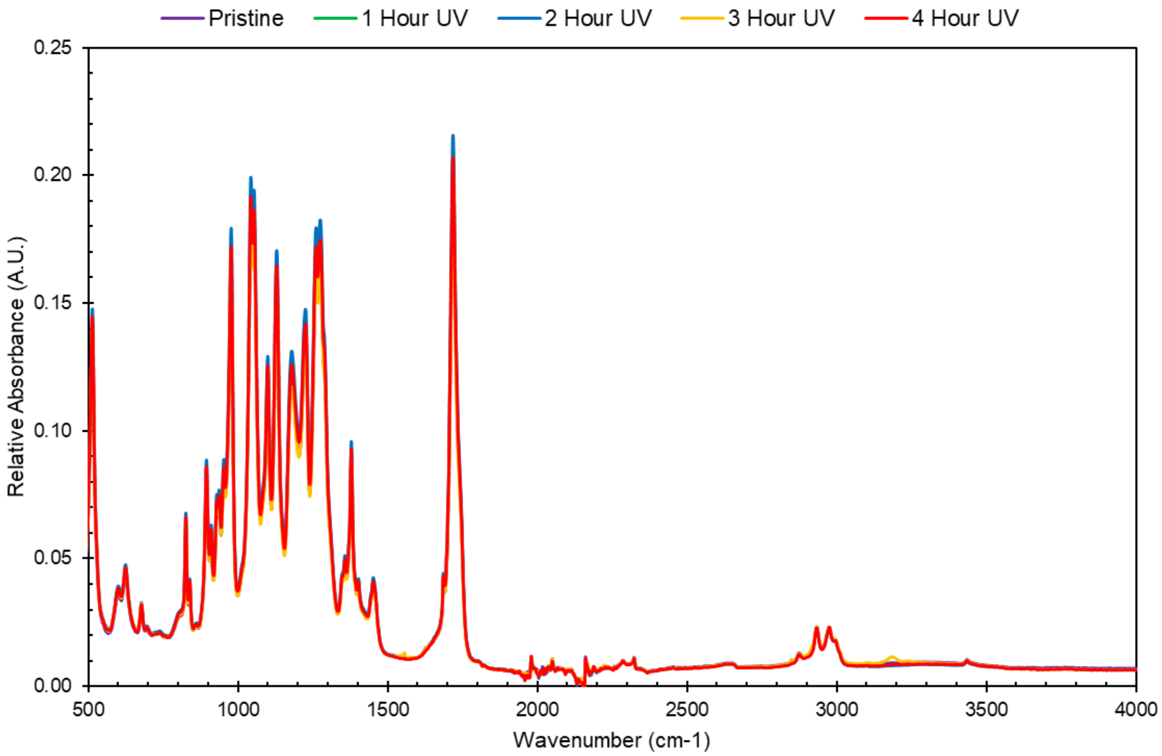

Figure S27. Averaged PHBV FTIR spectra by polymer irradiation time. Samples received enzymatic pre-treatment of 12 h in ~21 μM lipase. Samples ran in duplicate and averaged.

References

1. Wu, C.-S.; Wu, C.-T.; Yang, Y.-S.; Ko, F.-H. An Enzymatic Kinetics Investigation into the Significantly Enhanced Activity of Functionalized Gold Nanoparticles. *Chem. Commun.* **2008**, No. 42, 5327. https://doi.org/10.1039/b810889g.
2. Brown, M. H.; Badzinski, T. D.; Pardoe, E.; Ehlebracht, M.; Maurer-Jones, M. A. UV Light Degradation of Polylactic Acid Kickstarts Enzymatic Hydrolysis. *ACS Mater. Au* **2024**, *4* (1), 92–98. https://doi.org/10.1021/acsmaterialsau.3c00065.
3. Pérez, J. M.; Ruiz, C.; Fernández, I. Synthesis of a Biodegradable PLA: NMR Signal Deconvolution and End-Group Analysis. *J. Chem. Educ.* **2022**, *99* (2), 1000–1007. https://doi.org/10.1021/acs.jchemed.1c00824.
